# Calculation of Single Cell Assimilation Rates From SIP-NanoSIMS-Derived Isotope Ratios: A Comprehensive Approach

**DOI:** 10.3389/fmicb.2018.02342

**Published:** 2018-10-03

**Authors:** Hryhoriy Stryhanyuk, Federica Calabrese, Steffen Kümmel, Florin Musat, Hans H. Richnow, Niculina Musat

**Affiliations:** Department of Isotope Biogeochemistry, Helmholtz Centre for Environmental Research-UFZ, Leipzig, Germany

**Keywords:** nanoSIMS, single cell, assimilation rate, functional heterogeneity, stable isotope probing, isotope ratio, isotope fractionation

## Abstract

The nanoSIMS-based chemical microscopy has been introduced in biology over a decade ago. The spatial distribution of elements and isotopes analyzed by nanoSIMS can be used to reconstruct images of biological samples with a resolution down to tens of nanometers, and can be also interpreted quantitatively. Currently, a unified approach for calculation of single cell assimilation rates from nanoSIMS-derived changes in isotope ratios is missing. Here we present a comprehensive concept of assimilation rate calculation with a rigorous mathematical model based on quantitative evaluation of nanoSIMS-derived isotope ratios. We provide a detailed description of data acquisition and treatment, including the selection and accumulation of nanoSIMS scans, defining regions of interest and extraction of isotope ratios. Next, we present alternative methods to determine the cellular volume and the density of the element under scrutiny. Finally, to compensate for alterations of original isotopic ratios, our model considers corrections for sample preparation methods (e.g., air dry, chemical fixation, permeabilization, hybridization), and when known, for the stable isotope fractionation associated with utilization of defined growth substrates. As proof of concept we implemented this protocol to quantify the assimilation of ^13^C-labeled glucose by single cells of *Pseudomonas putida*. In addition, we provide a calculation template where all protocol-derived formulas are directly available to facilitate routine assimilation rate calculations by nanoSIMS users.

## Introduction

The use of nanoSIMS as a single-cell technique in environmental microbiology and microbial ecology has been enhanced over the past years (Milucka et al., 2012; Berry et al., 2013; Koch et al., 2014; Woebken et al., 2014; McGlynn et al., 2015; Martínez-Pérez et al., 2016; Oswald et al., 2017; Raina et al., 2017). A great number of studies employ the use of stable isotope labeling and nanoSIMS to track metabolic processes of single cells, *in situ*, in environmental and synthetic systems (Pett-Ridge and Weber, 2012; Musat N. et al., 2016). Thus, changes in isotope composition of single cells upon biotic assimilation of the stable isotope labeled growth substrate are measured by the nanoSIMS and further used quantitatively (e.g., (Musat et al., 2008; Thompson et al., 2012; Martínez-Pérez et al., 2016). Most studies employing the nanoSIMS combination with stable isotope probing (nanoSIP) have presented the isotope ratio values (Lechene et al., 2006, 2007), isotope enrichment values as atomic % excess or in delta notation (Popa et al., 2007; Fike et al., 2008; Dekas et al., 2009; Morono et al., 2011; Tourna et al., 2011; Thompson et al., 2012; Woebken et al., 2012; Berry et al., 2013; Lee et al., 2014; McGlynn et al., 2015) and some used the nanoSIMS-derived isotope ratios to quantify single-cell assimilation (Popa et al., 2007; Musat et al., 2008; Krupke et al., 2013, 2015; Martínez-Pérez et al., 2016; Schreiber et al., 2016; Nikolic et al., 2017; Zimmermann et al., 2018). In order to acquire accurate isotope ratios with nanoSIMS, required to calculate single cell assimilation rates with a great confidence, the following steps need to be carefully considered: processing of nanoSIMS data (e.g., selection of the optimal number of scans to be accumulated), proper identification of single cells, and accurate definition and selection of regions of interest (RoIs) around single cells. Finally, the calculations should consider the cellular biovolume and elemental density (e.g., cellular density of carbon or nitrogen), corrections for isotope dilution by various treatments during sample preparation (Musat et al., 2014; Woebken et al., 2014; Pernice et al., 2015; Musat N. et al., 2016). and, when known, corrections for compound-specific stable isotope fractionation during assimilation of labeled substrates (Elsner et al., 2005; Musat F. et al., 2016).

A comprehensive protocol to obtain assimilation rates of single cells from nanoSIMS-derived isotopic ratios and consensus formulas that can be applied by the scientific community on a general base are presently missing. The available calculation approaches based on empirical expressions for assimilation and growth rate differ considerably between nanoSIMS working groups, leading to difficulties in choosing the appropriate mathematical formulas to analyse particular samples with high confidence. The nanoSIP-based computation method reported for quantitation of single cell activity in chemostats (Kopf et al., 2015) implies a series of intermediate numerical calculations limiting the possibility to derive a generalized analytical expression for single cell assimilation applicable to other cell cultivation systems. Here we present a protocol where we explain step by step how one can use the isotopic ratios measured by nanoSIMS to calculate meaningful element-specific assimilation rates, what other parameters have to be considered when such calculations are applied, including how to account for the label dilution due to chemical treatments like fixation, dehydration, embedding, or hybridization steps. As a proof of concept, we further demonstrate how to use this protocol to quantify single cell assimilation rate on a set of data obtained by nanoSIMS analysis of *P. putida* culture incubated in the presence of ^13^C-glucose.

## Materials and methods

### Chemicals, organisms, and cultivation conditions

^13^C_6_-glucose was purchased from Chemotrade (Düsseldorf, Germany). *Pseudomonas putida* KT2440 (DSM6125) was routinely cultivated in 250 ml flasks containing 100 ml defined salt medium with glucose as growth substrate (1 g·l^−1^), as previously described (Musat et al., 2014). The bottles were inoculated with 5 ml of a culture in mid-exponential growth phase. Labeling experiments were conducted in 100 ml serum bottles with 66.5 ml mineral medium, 3.5 ml inoculum, 9.5 mg ^13^C_6_-labeled and 66 mg unlabeled glucose resulting in 13.5 at% labeling of the growth substrate with ^13^C isotope. To prevent transfer of unlabeled substrate with the inoculum, a volume of 10 ml was collected from a culture in the mid-exponential growth phase. The cells were collected by centrifugation, washed twice with 5 ml mineral medium devoid of carbon and nitrogen sources, and finally suspended in 3.5 ml mineral medium. The bottles were incubated in the dark at 30°C with horizontal shaking (200 rpm). Samples (20 ml) were collected after 10 h of incubation during the mid-exponential growth phase, and fixed for 2 h at room temperature with 2% v/v paraformaldehyde in 1 × PBS. Fixed cells were washed twice with deionized water, and suspended in 1 ml ethanol 50% v/v in deionized water. Volumes of 10 μl of fixed cells suspension were filtered on Au-Pd coated GTTP filters (Millipore, Eschborn, Germany; 25 mm diameter, 0.22 μm pore size), air dried and stored in vacuum at room temperature until nanoSIMS analysis.

### Nano-focused secondary ion mass spectrometry (NanoSIMS)

For the quantitative analysis of carbon assimilation rates the cells of *P. putida* were analyzed with a NanoSIMS-50 L instrument (CAMECA, AMETEK) in negative extraction mode employing a DC source of primary Cs^+^ ions. Implantation of cesium was done via presputtering of 80 × 80 μm^2^ sample areas with 0.15 nA of 16 keV Cs^+^ beam for 5 min with the purpose to stabilize the working function for negative secondary ions. The 4 pA beam of 16 keV Cs^+^ ions was focused into about 80 nm spot at the sample surface during the analysis. The sample was scanned in 256 × 256 pixels raster over 40 × 40 μm^2^ of presputtered area with 40 ms dwell time per pixel. The secondary ions were analyzed with double-focusing magnetic sector mass spectrometers for their mass-to-charge ratio (m/z) and detected in seven available collectors set for the following ion species: ^12^C^−^ (collector-1), ^13^C^−^ (collector-2), ^16^O^−^ (collector-3), ^12^C^14^N^−^ (collector-4), ^13^C^14^N^−^ (collector-5), ^12^C^16^O^−^ (collector-6), ^13^C^16^O^−^ (collector-7). The mass resolving power (MRP) was checked to be between 7,000 and 9,000 with the exit slit width of 100, 20 μm wide entrance slit, 200 μm aperture slit, and with the energy slit cutting 20% of secondary ions in high-energy tail of their energy distribution. The analyzed microbial cells were almost entirely sputtered within 8 scans upon the analysis conditions used and the scans 1–6 were considered for the analysis employing LANS software (Polerecky et al., 2012) allowing for the dead-time correction, accumulation of scanned planes with the lateral drift correction, definition of RoIs (Regions of Interest) for quantitative analysis of carbon isotope ratios (^13^C/^12^C, ^13^C^14^N/^12^C^14^N, and ^13^C^16^O/^12^C^16^O) explained in the description of results presented below.

### Determination of carbon density for *P. putida* cells

To determine the cellular carbon content of *P. putida* cells in fg·cell^−1^, their total carbon content measured with elemental analyzer was divided by the number of analyzed cells obtained by cell counting using a fluorescence microscope. The cellular carbon content was divided further by cell volume to get the final carbon-specific density of cell in fg·μm^−^^3^. For this calculation, the measurements of total carbon content, cell counting, and determination of cell volumes were performed as described below.

#### Measurement of total carbon content

*P. putida* cells were cultivated in 250 ml flasks provided with 200 ml culture media. Cultures were incubated as described above. Cells were collected at four different time points that fully encompass the initial and exponential growth phases (0, 4, 6, and 8 h). Culture volumes of 5 ml were filtered onto pre-combusted (450°C for 5 h) GF/F filters (25 mm diameter, Whatman™,GE Healthcare) using a vacuum filtration manifold device (Millipore® model 1225). Cells were washed three times with 5 ml of deionized water and dried at room temperature for 10 min. The filters were decalcified by incubation in an desiccator with 20% v/v HCl overnight. Round pieces of 5 mm diameter were cut out from the GFF filters using a hollow punch-out tool and packed in tin cups (HEKAtech GmbH, Germany). The filters were analyzed for their total carbon content with an EuroEA3000 elemental analyser (HEKAtech, Germany) in which the samples were completely combusted to CO_2_. The flash combustion was performed with a 10 mL O_2_ pulse in a combustion reactor filled with wolfram oxide and silver cobalt oxide (HE46820995, HEKAtech, Germany) at 1,050°C. Conversion products were separated on a packed GC-column (HE 26070500, HEKAtech, Germany) and transferred with helium as carrier gas via a ConFlo IV open split system to a MAT 253 IRMS (Thermo Fisher, Bremen). For quantification, a multi-point calibration with known amounts of sucrose was done.

#### Cell counting and calculation of cellular carbon content

For cell counting, volumes of 1 ml were collected from the same cultures and time points as above, and fixed for 2 h with 2% paraformaldehyde in 1 × PBS at 4°C. Volumes of 100 μl of the fixed cells suspension were diluted in 5 ml of PBS, filtered onto polycarbonate filters (25 mm diameter) using a vacuum filtration manifold device (Millipore® model 1225), washed once with PBS, once with deionized water and once with each of the following ethanol concentrations: 50, 70, and 80% v/v in deionized water. Filters were air dried for 15 min, stained with 4′,6-diamidine-2′-phenylindol (DAPI) dissolved in ultrapure water (1 μg·ml^−1^) for 10 min, washed twice with deionized water and once with 80% v/v ethanol. DAPI-stained filters were air dried for 15 min in the dark and mounted on glass slides with Citifluor/VectaShield (4:1). For each culture time point the cells from 10 fields of view were counted under a fluorescence microscope (Axio Imager.Z2, Carl Zeiss). The cell number counted within 87 × 67 μm^2^ field of view (FoV) was scaled up to the area of filter piece of 5 mm diameter to get the cell number analyzed for carbon content with the elemental analyzer. The division of total carbon content by the resulted cell number yielded a carbon cellular content of 277 ± 47 fg·cell^−1^ for the analyzed *P. putida* cells.

#### Determination of biovolume for *P. putida* cells

To derive the carbon cellular content per volume unit, the biovolume of *P. putida* cells was determined. *P. putida* cells were harvested in mid-exponential growth phase and fixed with 1% v/v glutaraldehyde (GA) in cacodylate buffer for 2 h at room temperature. Fixed cells were transferred on GTTP filters coated with a 30 nm layer of Au-Pd with the help of a stainless steel syringe filter holder (Sartorius, Germany), washed twice with 1 ml cacodylate buffer and post-fixed in KMnO_4_ (1% w/v in deionized water) at room temperature for 90 min. Filters were washed once with deionized water, followed by dehydration in an ethanol series of 30, 50, 70, 80, 90, 96, and 100% (3 min each). Subsequently, the filters were dried with a critical point dryer machine (Leica EM CPD 300a). The cells were observed and imaged with a scanning electron microscope (Merlin VP Compact, Carl Zeiss). Example of a SEM image is shown in Supplementary Material 1.1. The length (1.04 ± 0.14 μm) and width (0.57 ± 0.04 μm) of the cells measured with ImageJ on the acquired SEM images were used to calculate the biovolume of single cells with Equation (13). The obtained values of cell biovolume (0.22 ± 0.06 μm^3^) and the cellular carbon content derived from elemental analysis and cell counting resulted in the carbon cellular density of 1.27 ± 0.22 pg·μm^−3^.

Different chemical fixations of *P. putida* cells were considered for SEM imaging and nanoSIMS analysis. Preservation of cell morphology with GA was found to be superior to PFA fixation and was therefore applied in cell preparation for SEM imaging performed to determine the cell size for biovolume and elemental density calculations. We tested comparatively the cell fixation with GA and PFA (data are not shown). The best preservation of the cell morphology was achieved upon fixation with 1% GA. Instead, when preparing cells for the nanoSIMS analysis, we used a 2% PFA fixative, similar to fixation employed for CARD-FISH and fluorescence microscopy, since quite often these techniques are applied on environmental samples prior to and in combination with the nanoSIMS analysis. In addition, by applying the 2% PFA fixative we were consistent with our previous work (Musat et al., 2014) where the same concentration of PFA was used in chemical fixation of cells for the nanoSIMS analysis.

## Results and discussion

### Mathematical model to calculate assimilation rates

#### Calculation of assimilated elemental fraction from changes in isotope ratios

The fraction *K*_*A*_ of an element (e.g., carbon or nitrogen) assimilated by a cell during growth with isotope-labeled substrates can be evaluated from the changes in the cell's isotopic composition.

(1) $$\begin{array}{r} K_{A}=\frac{E_{a}}{E_{i}} \end{array}$$

*E*_*a*_ – amount of assimilated element,

*E*_*i*_ – initial cellular amount of the same element.

In the case of isotope ratio (*R*) defined as heavy-to-light ratio for carbon isotopes

R=C13C12

the fractions of heavy and light isotopes (*D*_*heavy*_ and *D*_*light*_) can be expressed as following.

Dheavy≡D=C13C13+C12=RR+1            Dlight=C12C13+C12=1R+1

The amounts of heavy and light isotopes (*E*_*Heavy*_ = *E*_*H*_ and *E*_*Light*_ = *E*_*L*_) in cells after incubation with isotope-labeled growth substrates can be expressed as a function of isotope ratios (*R*), initial and assimilated amount of an element (*E*_*i*_ and *E*_*a*_ as in Equation 1). For simplicity, in this first approach the assimilation of an element is considered as the mixing of two components with different isotopic composition (the cell and the culture media) as applied elsewhere (Popa et al., 2007).

(2) $$\begin{array}{r} E_{H}=E_{i}\times \frac{R_{i}}{R_{i}+1}+E_{a}\times \frac{R_{gs}}{R_{gs}+1} \end{array}$$

(3) $$\begin{array}{r} E_{L}=E_{i}\times \frac{1}{R_{i}+1}+E_{a}\times \frac{1}{R_{gs}+1} \end{array}$$

*R*_*gs*_—isotope ratio of growth substrate during incubation;

*R*_*i*_—initial cellular isotope ratio before incubation.

The isotope ratio after incubation (*R*_*f*_) can be expressed as ratio between heavy and light isotope amounts

$$\begin{array}{l} R_{f}=\frac{E_{H}}{E_{L}} \end{array}$$

Taking the Equations (2) and (3) into account

(4) $$\begin{array}{r} R_{f}=\frac{E_{i}\times \frac{R_{i}}{R_{i}+1}+E_{a}\times \frac{R_{gs}}{R_{gs}+1}}{E_{i}\times \frac{1}{R_{i}+1}+E_{a}\times \frac{1}{R_{gs}+1}} \end{array}$$

Division of the nominator and denominator of (4) by *E*_*i*_ will bring

(4') $$\begin{array}{l} R_{f}=\frac{\frac{R_{i}}{R_{i}+1}+\frac{E_{a}}{E_{i}}\times \frac{R_{gs}}{R_{gs}+1}}{\frac{1}{R_{i}+1}+\frac{E_{a}}{E_{i}}\times \frac{1}{R_{gs}+1}} \end{array}$$

Further transformations of Equation (4') yield the expression for *K*_*A*_.

(5) $$\begin{array}{r} \frac{R_{i}}{R_{i}+1}+\frac{E_{a}}{E_{i}}\times \frac{R_{gs}}{R_{gs}+1}=\frac{R_{f}}{R_{i}+1}+\frac{E_{a}}{E_{i}}\times \frac{R_{f}}{R_{gs}+1}\\ \frac{E_{a}}{E_{i}}\times \frac{R_{gs}}{R_{gs}+1}-\frac{E_{a}}{E_{i}}\times \frac{R_{f}}{R_{gs}+1}=\frac{R_{f}}{R_{i}+1}-\frac{R_{i}}{R_{i}+1}\\ \frac{E_{a}}{E_{i}}\times \left(\frac{R_{gs}-R_{f}}{R_{gs}+1}\right)=\frac{R_{f}-R_{i}}{R_{i}+1}\\ K_{A}=\frac{E_{a}}{E_{i}}=\frac{R_{f}-R_{i}}{R_{i}+1}\times \frac{R_{gs}+1}{R_{gs}-R_{f}} \end{array}$$

*K*_*A*_ can be finally expressed as a function of the initial and final isotope ratios of cells and the fraction *D*_*gs*_ of the heavy isotope in the growth substrate.

(6) $$\begin{array}{r} K_{A}=\frac{R_{f}-R_{i}}{R_{i}+1}\times \frac{R_{gs}+1}{R_{gs}-R_{f}}=\frac{R_{f}R_{gs}+R_{f}-R_{i}R_{gs}-R_{i}}{R_{i}R_{gs}-R_{i}R_{f}+R_{gs}-R_{f}}\\ =\frac{R_{gs}\left(R_{f}-R_{i}\right)+\left(R_{f}-R_{i}\right)}{R_{gs}\left(R_{i}+1\right)-R_{f}\left(R_{i}+1\right)}\\ K_{A}=\frac{\left(R_{f}-R_{i}\right)\times \left(R_{gs}+1\right)}{R_{gs}\left(R_{i}+1\right)-R_{f}\left(R_{i}+1\right)}\\ =\frac{R_{f}-R_{i}}{D_{gs}\left(R_{i}+1\right)-\frac{R_{f}\left(R_{i}+1\right)}{R_{gs}+1}}\\ K_{A}=\frac{R_{f}-R_{i}}{\left(R_{i}+1\right)\times \left(D_{gs}-\frac{R_{f}}{R_{gs}+1}\right)}\\ \frac{1}{R_{gs}+1}=\frac{R_{gs}+1-R_{gs}}{R_{gs}+1}=\frac{R_{gs}+1}{R_{gs}+1}-\frac{R_{gs}}{R_{gs}+1}=1-D_{gs}\\ K_{A}=\frac{R_{f}-R_{i}}{\left(1+R_{i}\right)\times \left(D_{gs}\times \left(1+R_{f}\right)-R_{f}\right)} \end{array}$$

Equation (6) expresses the fraction *K*_*A*_ of the element incorporated by a cell during incubation with isotope-labeled growth substrates, relative to its initial cellular content.

Usually, the cellular isotope ratios measured by nanoSIMS are directly used as the initial and final cellular isotope ratios (*R*_*i*_*, R*_*f*_ in Equation 6). In such cases, calculation of *K*_*A*_ does not account for inherent alterations of isotopic composition due to sample processing prior to the nanoSIMS analyses. However, in most studies, biological samples are subjected to various treatments following incubations with stable isotope labeled substrates. In the following section, we present a concept to restore cellular isotope ratios after sample treatments.

#### Restoration of isotope composition after chemical treatments

Preparation of biological samples for nanoSIMS analyses range from sample dehydration to meet the high vacuum analytical conditions of the nanoSIMS instrument, to metabolic inactivation, chemical fixation or cell wall permeabilization. Typical agents include aldehyde based compounds (e.g., formaldehyde, glutaraldehyde) or alcohols (e.g., ethanol, methanol). In addition, in many microbial ecology studies, cell phylogenetic identification is desired and chemical fixation is followed by fluorescence *in situ* hybridization based protocols such as FISH or CARD-FISH. This is increasing considerably the number of chemicals applied on the samples, and may significantly alter the isotopic composition. For example, we showed that chemical fixation and hybridization strongly affects the carbon and nitrogen isotope composition of microbial cells (Musat et al., 2014). Such changes of carbon isotope composition are mainly due to introduction of carbon from chemicals possessing natural isotope composition (~1.1 at% of ^13^C) into ^13^C-enriched microbial cells causing the dilution of ^13^C label. Such dilution effects have to be considered for the evaluation of element assimilation rates.

The original isotope ratios *R* of cells before chemical treatment can be restored from the isotope ratios *R'* derived after nanoSIMS experiment on chemically treated cells. For such a restoration, the fraction (*K*) of an element introduced into microbial cells upon chemical treatment and the *D*_*ch*_ fraction of heavy isotope of the applied chemicals have to be considered.

The fraction *K* of an element introduced into microbial cells upon chemical treatments can be defined relative to (i) an initial element content (*E*_*i*_) in cells before treatment or (ii) final element content (*E*_*f*_) including the element amount (*E*_*ch*_) introduced into cells upon chemical treatment. The *K* fraction is expressed as *K*_*i*_ relative to *E*_*i*_ and as *K*_*f*_ relative to *E*_*f*_ in the following way.

$$\begin{array}{l} K_{i}=\frac{E_{ch}}{E_{i}};\;K_{f}=\frac{E_{ch}}{E_{f}}=\frac{E_{ch}}{E_{i}+E_{ch}} \end{array}$$

The value of *K*_*i*_ has been derived from the carbon isotope ratio *R'* measured for *P. putida* cells grown on medium with 100% ^13^C-labeled glucose as carbon source after applying different chemical treatments (Musat et al., 2014).

(7) $$\begin{array}{r} K_{i}=\frac{R-R^{{\;}^{\prime }}}{\left(1+R\right)\times \left(R^{{\;}^{\prime }}-D_{ch}\times \left(R^{{\;}^{\prime }}+1\right)\right)} \end{array}$$

where: *R*—original cellular isotope ratios before chemical treatment;

*R*′—nanoSIMS measured isotope ratios after chemical treatment;

*D*_*ch*_—fraction of ^13^C isotope of the chemicals applied.

These *K*_*i*_ values can be cautiously applied to similar microorganisms or derived specifically for other types of microbial cells and chemical treatments in the same way (Musat et al., 2014). Other *K* values were determined for *Vibrio cholerae, Bacillus subtilis, E. coli* and *Deltaproteobacteria* from microbial mats (Woebken et al., 2014; Musat N. et al., 2016). When choosing an appropriate *K* value for the restoration of original isotope ratios, one should consider the difference and the following relation between *K*_*i*_ and *K*_*f*_ fractions.

(7') $$\begin{array}{ll} K_{i} & =\frac{K_{f}}{1-K_{f}};\Delta K_{i}=\frac{\partial K_{i}}{\partial K_{f}}\times \Delta K_{f}=\left(\frac{1}{1-K_{f}}+\frac{K_{f}}{{\left(1-K_{f}\right)}^{2}}\right)\times \Delta K_{f}\\ K_{f}=\frac{K_{i}}{1+K_{i}};\Delta K_{f}=\frac{\partial K_{f}}{\partial K_{i}}\times \Delta K_{i}=\left(\frac{1}{1+K_{i}}-\frac{K_{i}}{{\left(1+K_{i}\right)}^{2}}\right)\times \Delta K_{i}\\ \;K_{f}=\frac{R-R^{{\;}^{\prime }}}{\left(1+R^{{\;}^{\prime }}\right)\times \left(R-D_{ch}\times \left(R+1\right)\right)} \end{array}$$

With the measured *R*′ ratios and an appropriate *K*_*i*_ or *K*_*f*_ value, the original isotope ratios *R* can be calculated using one of the following expressions.

(8) $$\begin{array}{r} R=\frac{R^{{\;}^{\prime }}+K_{i}\times \left(R^{{\;}^{\prime }}-D_{ch}\times \left(R^{{\;}^{\prime }}+1\right)\right)}{1-K_{i}\times \left(R^{{\;}^{\prime }}-D_{ch}\times \left(R^{{\;}^{\prime }}+1\right)\right)} \end{array}$$

(8') $$\begin{array}{r} R=\frac{R^{{\;}^{\prime }}-K_{f}\times D_{ch}\times \left(R^{{\;}^{\prime }}+1\right)}{1-K_{f}\times \left(1+R^{{\;}^{\prime }}-D_{ch}\times \left(R^{{\;}^{\prime }}+1\right)\right)} \end{array}$$

The fraction of ^13^C isotope in chemicals (*D*_*ch*_) can be assumed to be 0.011 corresponding to the natural ^13^C abundance (1.1 at%) or it can be measured for specific chemicals to increase the accuracy of the calculation.

The *R* values expressed with (8) are to be used as final or initial isotope ratios (*R*_*f*_ or *R*_*i*_) for the calculation of the *K*_*A*_ fraction of carbon assimilated by the cells during their incubation in ^13^C labeled medium.

The error Δ*R* is calculated taking into account the uncertainties of input values (Δ*R*′, Δ*K*_*i*_, Δ*D*_*ch*)_ contributing in the error propagation (Fitzsimons et al., 2000).

$$\begin{array}{l} \Delta R=\sqrt{{\left(\frac{\partial R}{\partial R^{{\;}^{\prime }}}\times \Delta R^{{\;}^{\prime }}\right)}^{2}+{\left(\frac{\partial R}{\partial K_{i}}\times \Delta K_{i}\right)}^{2}+{\left(\frac{\partial R}{\partial D_{ch}}\times \Delta D_{ch}\right)}^{2}}\\ \frac{\partial R}{\partial R^{{\;}^{\prime }}}=\frac{\left(1+K_{i}\times \left(1-D_{ch}\right)\right)\times \left[1-K_{i}\times \left(R^{{\;}^{\prime }}-D_{ch}\times \left(R^{{\;}^{\prime }}+1\right)\right)\right]}{{\left[1-K_{i}\times \left(R^{{\;}^{\prime }}-D_{ch}\times \left(R^{{\;}^{\prime }}+1\right)\right)\right]}^{2}}+\\ +\frac{K_{i}\times \left(1-D_{ch}\right)\times \left[R^{{\;}^{\prime }}+K_{i}\times \left(R^{{\;}^{\prime }}-D_{ch}\times \left(R^{{\;}^{\prime }}+1\right)\right)\right]}{{\left[1-K_{i}\times \left(R^{{\;}^{\prime }}-D_{ch}\times \left(R^{{\;}^{\prime }}+1\right)\right)\right]}^{2}}\\ \frac{\partial R}{\partial K_{i}}=\frac{\left(R^{{\;}^{\prime }}+1\right)\times \left(R^{{\;}^{\prime }}-D_{ch}\times \left(R^{{\;}^{\prime }}-1\right)\right)}{{\left[1-K_{i}\times \left(R^{{\;}^{\prime }}-D_{ch}\times \left(R^{{\;}^{\prime }}+1\right)\right)\right]}^{2}}\\ \frac{\partial R}{\partial D_{ch}}=\frac{-K_{i}\times {\left(R^{{\;}^{\prime }}+1\right)}^{2}}{{\left[1-K_{i}\times \left(R^{{\;}^{\prime }}-D_{ch}\times \left(R^{{\;}^{\prime }}+1\right)\right)\right]}^{2}} \end{array}$$

For the correction of nitrogen isotope ratio with (8) the applicability of the dilution model has to be proved and the treatment-specific *K* values for nitrogen have to be derived or set to “0” if the reduction of ^15^N/^14^N ratio is not observed for the treatment applied. The *K* = 0 case implies the absence of isotope dilution and yields *R* = *R'* (8).

#### Consideration of stable isotope fractionation effects

Biochemical reactions usually discriminate against the heavy isotopes, i.e., preferential processing of lighter molecules of the growth substrate, leading to an accumulation of heavy isotopes in the residual substrate pool. This effect, described by the isotope fractionation factor α (10), may cause differences between the isotope ratio of assimilated substrate (*R*_*assim*_) and the respective ratio of the growth substrate (*R*_*gs*_). If the isotope fractionation factor α is known, it can be considered to refine the calculation of an element fraction assimilated from growth substrate (Equation 6).

In the expression of *K*_*A*_ (6) we will substitute the *D*_*gs*_ with a fraction of heavy isotope in assimilated substrate (*D*_*assim*_):

(9) $$\begin{array}{r} K_{A}=\frac{R_{f}-R_{i}}{\left(1+R_{i}\right)\times \left(D_{assim}\times \left(1+R_{f}\right)-R_{f}\right)} \end{array}$$

*D*_*assim*_ is expressed as a function of *R*_*gs*_ and α. With the *R*_*gs*_ and *R*_*assim*_ denotations, the isotope fractionation factor is defined as

(10) $$\begin{array}{r} \alpha_{gs/assim}=\frac{R_{gs}}{R_{assim}} \end{array}$$

The expression for the isotope fraction of assimilated substrate (*D*_*assim*_) is derived in following way.

According to Equation 10:

$$\begin{array}{l} R_{assim}=R_{gs}/\alpha \\ D_{assim}=\frac{R_{assim}}{R_{assim}+1}\\ D_{assim}=\frac{R_{gs}/\alpha }{R_{gs}/\alpha +1}=\frac{R_{gs}}{R_{gs}+\alpha } \end{array}$$

Considering the expression of *D*_*assim*_ in Equation 9 leads to:

(11) $$\begin{array}{r} K_{A}=\frac{R_{f}-R_{i}}{\left(1+R_{i}\right)\times \left[\frac{R_{gs}}{R_{gs}+\alpha }\times \left(1+R_{f}\right)-R_{f}\right]} \end{array}$$

Note that if the fractionation factor α is set to 1 (unknown for the organism or growth substrate under study), Equation 11 will revert to Equation 6.

The error Δ*K*_*A*_ is calculated taking into account the contribution of input value uncertainties (Δ*R*_*i*_, Δ*R*_*f*_, Δ*R*_*gs*_, Δα_)_ in the error propagation (Fitzsimons et al., 2000).

$$\begin{array}{l} \Delta K_{A}=\sqrt{{\left(\frac{\partial K_{A}}{\partial R_{i}}\times \Delta R_{i}\right)}^{2}+{\left(\frac{\partial K_{A}}{\partial R_{f}}\times \Delta R_{f}\right)}^{2}+{\left(\frac{\partial K_{A}}{\partial R_{gs}}\times \Delta R_{gs}\right)}^{2}+{\left(\frac{\partial K_{A}}{\partial \alpha }\times \Delta \alpha \right)}^{2}}\\ \frac{\partial K_{A}}{\partial R_{i}}=\frac{R_{f}+1}{{\left(R_{i}+1\right)}^{2}\times \left[R_{f}-\frac{R_{gs}}{R_{gs}+\alpha }\times \left(R_{f}+1\right)\right]}\\ \frac{\partial K_{A}}{\partial R_{f}}=\frac{\frac{R_{gs}}{R_{gs}+\alpha }\times \left(R_{i}+1\right)-R_{i}}{\left(R_{i}+1\right)\times {\left[R_{f}-\frac{R_{gs}}{R_{gs}+\alpha }\times \left(R_{f}+1\right)\right]}^{2}}\\ \frac{\partial K_{A}}{\partial R_{gs}}=\frac{1/\alpha \times \left({R_{i}}^{2}\times R_{f}-R_{i}\times {R_{f}}^{2}+{R_{i}}^{2}-{R_{f}}^{2}+R_{i}-R_{f}\right)}{{\left[\left(R_{i}+1\right)\times \left(R_{f}-\frac{R_{gs}}{R_{gs}+\alpha }\times \left(R_{f}+1\right)\right)\times \left(R_{gs}/\alpha +1\right)\right]}^{2}}\\ \frac{\partial K_{A}}{\partial \alpha }=\frac{R_{gs}\times \left({R_{i}}^{2}\times R_{f}-R_{i}\times {R_{f}}^{2}+{R_{i}}^{2}-{R_{f}}^{2}+R_{i}-R_{f}\right)}{{\left[\left(R_{i}+1\right)\times \left(R_{f}-\frac{R_{gs}}{R_{gs}+\alpha }\times \left(R_{f}+1\right)\right)\times \left(R_{gs}/\alpha +1\right)\right]}^{2}} \end{array}$$

#### Dynamics of K_A_ as a function of the final fraction D_f_ of an assimilated element

**Figure 1:**
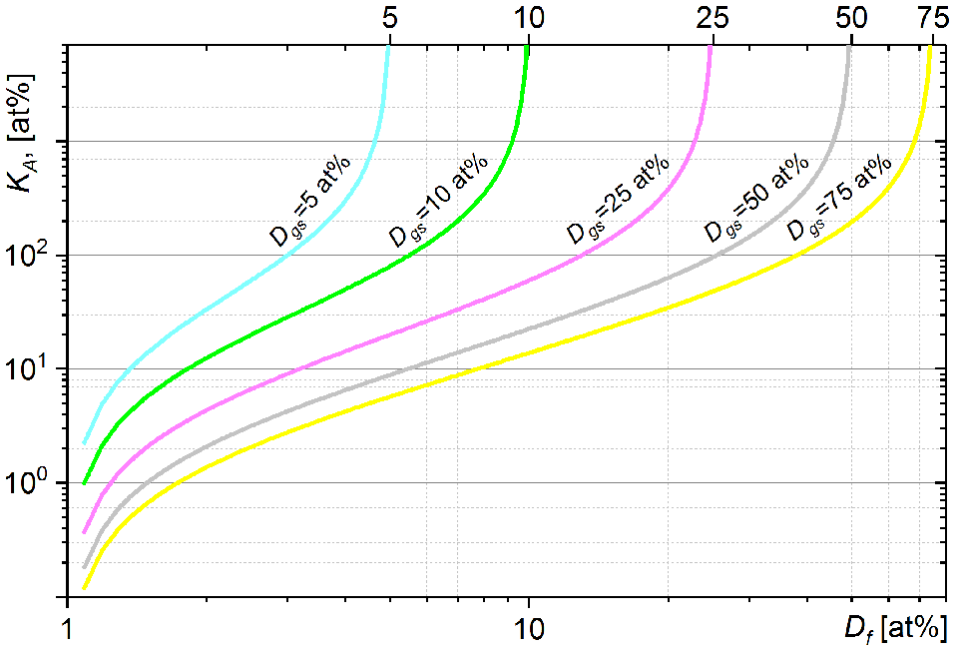
Dependence of assimilated carbon fraction *K*_*A*_ (11) on the final ^13^C fraction *D*_*f*_ simulated for the cells incubated from the inoculum with *D*_*i*_ = 1 at% in growth substrates with different *D*_*gs*_ values of ^13^C fraction.

The graphs in Figure 1 show the fraction of carbon (*K*_*A*_, Equation 11) which a cell of an initial (natural) carbon isotope composition (*D*_*i*_ = 1 at%) has to assimilate (relatively to an initial carbon cellular content) from growth substrate with ^13^C fraction *D*_*gs*_ to reach the final *D*_*f*_ cellular fraction of ^13^C. The *K*_*A*_ (*D*_*f*_) dependence shows the nonlinear relation between the assimilation and heavy isotope fraction. An increase in heavy isotope fraction (Δ*D*_*f*_) requires more labeled substrate to be assimilated (Δ*K*_*A*_) at higher *D*_*f*._ A *K*_*A*_ fraction value ≥ *n* × 100 at% means that it was achieved by a cell in *n* + 1 generation (after *n* divisions). The *K*_*A*_ dependences show asymptotic profiles limiting the cellular ^13^C fraction *D*_*f*_ below the ^13^C fraction in growth substrate *D*_*gs*_. The value of cellular ^13^C fraction *D*_*f*_ > *D*_*gs*_ can be achieved only with isotope fractionation factor α < 1 (see Equation 11). The comparison of *K*_*A*_ expression (Equation 11) with the expression of net assimilation reported by (Popa et al., 2007) is presented in Supplementary Material 2.

The *K*_*A*_(*D*_*f*_) dependence shows fast changes of *K*_*A*_ when *D*_*f*_ approaches the *D*_*gs*_. These fast changes of *K*_*A*_ are revealed in high absolute values of *K*_*A*_ derivatives expressed above and imply an increase in Δ*K*_*A*_ when *K*_*A*_ is calculated for *D*_*f*_ approaching the *D*_*gs*_. Uncertainty of *K*_*A*_ calculated with isotope fractionation factor neglected (α set to 1) or incorrectly set becomes also considerable with *D*_*f*_ approaching the *D*_*gs*_ (see Supplementary Material 1.2 and Figure S4). It can therefore be recommended to derive the *K*_*A*_ with *D*_*f*_ below 0.6 × *D*_*gs*_. In general, higher *D*_*gs*_ value reduces the uncertainty of *K*_*A*_ due to neglected or incorrect α value and provides a broader dynamic range for *K*_*A*_. It is therefore important to have sufficiently high value of *D*_*gs*_ especially for analysis of complex microbial communities when (i) isotope fractionation factor α has to be neglected (α set to 1) or set to an approximated value and (ii) different species reveal considerably different *K*_*A*_ values (assimilation) distributed in a broad range. On the other hand, (i) increase in heavy isotope content in growth substrate (*D*_*gs*_) may affect the metabolic processes in the studied microbial systems, (ii) extreme *D*_*gs*_ value yields poor counting rate for a light isotope.

#### Cell volume and element-specific cellular density

Besides *K*_*A*_ values describing relative assimilation, the expression of assimilation rate in mass units per time for each single cell (*see detailed implementation in* Supplementary Material Excel Template Table) requires also (i) a value of element-specific cell density (e.g., mass of carbon or nitrogen per cell volume) for expression of the volume-specific assimilation rate for each single cell in e.g., fg·μm^−3^·h^−1^ and (ii) volume of each single cell for expression of the cell-specific assimilation rate in e.g., fg·cell^−1^·h^−1^. Dispersion of cell volumes may be considerable even for cells in pure cultures (e.g., relative to their growth state).

##### Calculation of RoI-confined cellular volume from NanoSIMS data

Treatment of nanoSIMS data considering single-cells implies the definition of each single cell by drawing the RoIs confining single cells in nanoSIMS-acquired ion yield maps. Cellular volume confined by RoI can be estimated from the data of nanoSIMS experiment providing the area (Sp, given in pixels) of RoI defined around a single cell and the Length-to-Width Ratio of RoI (LWR). Very often RoI defined with a nanoSIMS map does not confine a single cell, but rather a cell fragment. The length (Lp) and width (Wp) of RoI-confined fragments of rod-shaped and coccoid cell can be expressed in pixels as following.

$$\begin{array}{l} \{\begin{array}{ll} \text{​}S_{p} & \text{​}=\text{​}\left(L_{p}-W_{p}\right)\times W_{p}\text{​}+\text{​}\frac{\pi }{4}W_{p}{}^{2}\\ \text{​}L_{p} & \text{​}=\text{​}LWR\times W_{p} \end{array}\Rightarrow S_{p}=W_{p}{}^{2}\times \left(LWR-1+\frac{\pi }{4}\right)\\ \;W_{p}=\sqrt{\frac{S_{p}}{LWR-1+\frac{\pi }{4}}}\\ \;L_{p}=\sqrt{\frac{S_{p}}{LWR-1+\frac{\pi }{4}}}\times LWR \end{array}$$

The raster metric dimension (length of rectangular raster, *FoV* [μm]) and the **R**a**st**er size in pixels [number of pixels along the raster edge, *Rst* [pixel]] can be used to convert *L*_*p*_ and *W*_*p*_ into corresponding *L* and *W* values in metric scale.

(12) $$\begin{array}{r} \begin{array}{c} L=L_{p}\times FoV/Rst=\sqrt{\frac{S_{p}}{LWR-1+\frac{\pi }{4}}}\times LWR\times FoV/Rst\\ W=W_{p}\times FoV/Rst=\sqrt{\frac{S_{p}}{LWR-1+\frac{\pi }{4}}}\times FoV/Rst \end{array} \end{array}$$

For example, for rod-shaped cells the biovolume (*V*) can be calculated as the sum of a cylinder of *W* μm in diameter and (*L–W*) μm in length, capped on it's both sides with hemispheres of *W* μm in diameter:

$$\begin{array}{l} V_{cyl}=\pi \frac{W^{2}}{4}\times \left(L-W\right) \end{array}$$

and the volume of two capping hemispheres

$$\begin{array}{l} V_{sph}=\frac{4}{3}\pi {\left(\frac{W}{2}\right)}^{3}=\frac{1}{6}\pi \times W^{3} \end{array}$$

in the following way

(13) $$\begin{array}{r} V=\frac{1}{2}\pi \times W^{2}\times \left(\frac{1}{3}W+\frac{1}{2}\left(L-W\right)\right) \end{array}$$

Note that if *L* = *W*, equation 13 will express the volume of a sphere, so it can be also used to calculate the biovolume of coccoid cells. Expression (13) can also be applied to calculate cellular volumes when *L* and *W* are derived from other analyses, e.g., Scanning Electron Microscopy (SEM) or Atomic-Force Microscopy (AFM), as well as for the calculation of volume for ROI-confined cell fragments considering the values of *L* and *W* derived from the nanoSIMS data according to expressions (12). For the measurements of cell volume a series of cautions has to be obeyed upon different treatments (e.g., fixation, dehydration, and preparation for Ultra-High Vacuum (UHV) sample environment of SEM, SIMS etc.) in order to avoid any distortion of cell native geometry due to cell expansion/burst, shrinkage, cell agglomeration etc.

##### Calculation of cellular element-specific density

An absolute value of elemental (carbon, nitrogen etc.) content per cell volume (partial density of e.g., carbon: ρC [g/μm^3^]) can be expressed in terms of (i) total amount of an element per sample [e.g., MC [g] of carbon]; (ii) number of cells per sample (N); and (iii) averaged cell volume (V) in the following way.

(14) $$\begin{array}{r} \rho_{C}=\frac{M_{C}}{N\times V} \end{array}$$

Total absolute amount of an element per sample (M_C_ [g]) can be derived from an Elemental Analysis Mass Spectrometry (EA-MS) experiment.

Number of cells per sample (*N*) can be determined by flow cytometry or, depending on the size of the target cells, by direct counting under a haemocytometer. Both methods are most effective if interferences from other particles (e.g., detritus, sediment) are not expected, and hence amenable to relatively clean samples like pure or enriched cultures, or environmental water samples. Alternatively, cells can be stained with nucleic acid dyes (e.g., DAPI) and counted under a epifluorescence microscope, a method that can be applied to most types of samples.

Cell volume (*V*) can be calculated using the expression (13) with cell length and width values measured using ImageJ on SEM images of cells as mentioned in the section Determination of Biovolume for *P. putida* Cells. Note that the mean value of cell volume (*V*) and its standard deviation derived from SEM images are used here for calculation of cellular element-specific density (e.g., ρ_*C*_) only and should not be confused with the RoI-confined cellular volumes (*V*_*i*_) derived from nanoSIMS data for each single cell analyzed to calculate the cell-specific assimilation rate (see section Cell-Specific Assimilation Rate).

If EA-MS or Flow Cytometry are not available, the element-specific cell density can be calculated as follows.

- Estimate the cell dry mass (*DM*) from cell volume (*V*) within the approach developed by Loferer-Krössbacher et al. (1998)
  (15) $$\begin{array}{r} DM=435\times V^{0.86} \end{array}$$
- Calculate an element-specific content for a single cell (e.g., *m*_*C*_ of carbon) from the total dry mass of cell (*DM*) using Redfield elemental ratio of cell composition (Fagerbakke et al., 1996). Note that this approach may introduce uncertainties since the cellular elemental ratios may deviate from the Redfield ratio among different phylogenetic groups, or may be influenced by the type, growth conditions or availability of nutrients.
- Calculate an element-specific cell density e.g., for carbon
  (16) $$\begin{array}{r} \rho_{C}=\frac{m_{C}}{V};m_{C}=\frac{M_{C}}{N} \end{array}$$

Considering the molar masses (μ_*C*_ and μ_*N*_) and the Redfield ratio, e.g., for atomic composition of marine phytoplankton

(17) $$\begin{array}{r} \left\{\nu_{P},\nu_{N},\nu_{C}\right\}=\left\{1,16,106\right\} \end{array}$$

the element-specific partial densities (ρ_*C*_ and ρ_*N*_) can be linked with the following expression.

(18) $$\begin{array}{r} \rho_{N}=\frac{\nu_{N}}{\nu_{C}}\times \frac{\mu_{N}}{\mu_{C}}\times \rho_{C} \end{array}$$

With the values from Redfield ratio sequence (17) and respective molar masses Equation 18 yields

(19) $$\begin{array}{r} \rho_{N}=\frac{16}{106}\times \frac{14}{12}\times \rho_{C} \end{array}$$

The cellular density of major elements, particularly carbon, has been experimentally determined for phylogenetically and morphologically diverse microorganisms (Table 1). Such values could be cautiously considered for phylogenetically and physiologically related strains if any of the required parameters to calculate the density of an element for cells of interest is missing.

**Table 1:** Carbon density/content and cell biovolume for a selection of microorganisms from various phylogenetic clades.

**Strain**	**Cell volume [μm^3^]**	**Carbon density [fg·μm^−3^]**	**Carbon content [fg ·cell^−1^]**	**References**
***Alphaproteobacteria***				
*Ca*. Pelagibacter			32.2	Zimmerman et al., 2014
*Oceanicola granulosus*			94.8	Zimmerman et al., 2014
*Ruegeria pomeroyi*			142	Zimmerman et al., 2014
*Pelagibaca bermudensis*			172	Zimmerman et al., 2014
***Gammaproteobacteria***				
*Cycloclasticus oligotrophus*	0.26 ± 0.02		9.46 ± 3.64	Robertson et al., 1998
*Vibrio campbellii*	0.21^*^		14.51 ± 3.48	Troussellier et al., 1997
*Enterobacter cloacae*	0.27^*^		18.57 ± 2.44	Troussellier et al., 1997
*Salmonella typhimurium*	0.57^*^		24.07 ± 3.00	Troussellier et al., 1997
*Escherichia coli*	0.71^*^		24.96 ± 4.55	Troussellier et al., 1997
*Alteromonas rubra*	0.70^*^		29.96 ± 7.50	Troussellier et al., 1997
*Aeromonas hydrophyla*	0.64^*^		30.59 ± 2.84	Troussellier et al., 1997
*Alteromonas nigrifaciens*	0.34^*^		31.63 ± 4.20	Troussellier et al., 1997
*Alteromonas tetraodonis*	0.37^*^		33.26 ± 6.09	Troussellier et al., 1997
*Vibrio fischeri*	0.22^*^		33.45 ± 5.36	Troussellier et al., 1997
*Pseudomonas putida KT2440*	0.22 ± 0.06	1269 ± 216	277.0 ± 47.0	This study
*Vibrio natriegens*	3.5 ± 0.5	350 ± 40		Fagerbakke et al., 1996
***Deltaproteobacteria***				
*Desulfosarcina* sp. strain BuS5	2.96	55.2	163.2	Jaekel et al., 2013
***Cyanobacteria***				
*Phormidium autumnale*		93.37 ± 3.27		Mahlmann et al., 2008
*Anabaena cf. thermalis*		118.84 ± 4.04		Mahlmann et al., 2008
*Spirulina platensis*		125.77 ± 3.72		Mahlmann et al., 2008
*Oscillatoria cf. rupicola*		129.26 ± 5.56		Mahlmann et al., 2008
*Prochlorococcus SARG*	0.144 ± 0.008	237 ± 7		Heldal et al., 2003
*Prochlorococcus SB*	0.22 ± 0.01	147 ± 5		Heldal et al., 2003
*Synechococcus WH 7803*	0.62 ± 0.07		120 ± 10	Heldal et al., 2003
*Synechococcus WH 8103*	0.83 ± 0.06		220 ± 10	Heldal et al., 2003
***Environmental species***				
*Bacterioplankton* Lake Slaen,Vermount USA	0.286 ± 0.027	210 ± 30		Bjørnsen, 1986
*Bacterioplankton* Lake Bryrup, Danemark	0.278 ± 0.042	340 ± 90		Bjørnsen, 1986
*Bacterioplankton* Roskilde Fjord, Danemark	0.101 ± 0.009	340 ± 10		Bjørnsen, 1986
*Bacterioplankton* Raunefjorden, Norway	1.78 ± 0.17		149 ± 8	Vrede et al., 2002

* *Calculated using Equation 13 (this study) with values of cell length and width from Table 1 in Troussellier et al. (1997)*.

### Calculation of assimilation rates

Rates of assimilation can be calculated for each single microbial cell by dividing the assimilated amount of an element (e.g., carbon or nitrogen) by the incubation time. The fraction of heavy isotope in growth substrate (*D*_*gs*)_ must be accepted to be stable (within a precision) during the considered time-frame of incubation. An incubation period may be split into several time-frames when strong changes in the availability or isotopic composition of the growth substrate are revealed/expected. Generalized scheme of assimilation rate studies is presented in Supplementary Material 3. The calculation procedure is implemented in Supplementary Material Excel Template Table.

#### Relative assimilation

The value of *K*_*A*_ (Equation 11) represents the fraction of an assimilated element relative to its initial content in a cell. The relative assimilation expressed with *K*_*A*_ values can be used in comparative studies for analysis of a relative difference and heterogeneity in single cell activity revealed in relative amount of assimilated material (in *K*_*A*_ values). If *K*_*A*_ values are derived at several time points, the slope in *K*_*A*_ (*t*) plot shows the trend of assimilation rate in time.

#### Cell-specific assimilation rate

Multiplying the absolute value of an element mass per cell volume (carbon partial density ρ_*C*_ [g/μm^3^], for example) by the RoI-confined cellular volume (*V*_*i*_ [μm^3^], *i* ∈ {1 … n}) calculated for each of *n* analyzed cells using (12) and (13) results in the mass (*m*_*i*_ [g], *i* ∈ {1 … n}) of carbon of each of the analyzed cells

(20) $$\begin{array}{r} m_{i}=\rho_{C}\times V_{i},i\in \left\{1\ldots n\right\} \end{array}$$

where *n* is the number of analyzed cells.

To derive the mass of carbon (*u*_*i*_) assimilated by each cell, the cell-specific mass of carbon (*m*_*i*_) has to be multiplied by the fraction *K*_*A*_ (11) of carbon incorporated into each cell.

(21) $$\begin{array}{r} u_{i}=m_{i}\times K_{A},i\in \left\{1\ldots n\right\} \end{array}$$

The cell-specific assimilation rate (*F*_*c*)_ is calculated for each cell by dividing the *u*_*i*_ over the incubation time (*t*).

(22) $$\begin{array}{r} F_{c}=\frac{u_{i}}{t}=\frac{\rho \times V_{i}\times K_{A}}{t},i\in \left\{1\ldots n\right\} \end{array}$$

The error Δ*F*_*c*_ is calculated taking into account the uncertainties of input values (Δρ, Δ*V*, Δ*K*_*A*_, Δ*t*) in the following way (Fitzsimons et al., 2000).

$$\begin{array}{l} \Delta F_{c}=\sqrt{{\left(\frac{\partial F_{c}}{\partial \rho }\times \Delta \rho \right)}^{2}+{\left(\frac{\partial F_{c}}{\partial V}\times \Delta V\right)}^{2}+{\left(\frac{\partial F_{c}}{\partial K_{A}}\times \Delta K_{A}\right)}^{2}+{\left(\frac{\partial F_{c}}{\partial t}\times \Delta t\right)}^{2}}\\ \frac{\partial F_{c}}{\partial \rho }=\frac{V\times K_{A}}{t};\frac{\partial F_{c}}{\partial V}=\frac{\rho \times K_{A}}{t};\frac{\partial F_{c}}{\partial K_{A}}=\frac{\rho \times V}{t};\frac{\partial F_{c}}{\partial t}=-\frac{\rho \times V\times K_{A}}{t^{2}}; \end{array}$$

#### Volume-specific assimilation rates

To eliminate the dispersion of *F*_*c*_ values due to the variation of RoI-confined cellular volume, the cell-specific assimilation rate (*F*_*c*)_ of each cell can be normalized by the RoI-confined volume (*V*_*i*__)_. This normalization is particularly important when isotopic ratios are derived for cell fragments confined within RoIs in isotope ratio maps acquired with nanoSIMS. The volume-specific assimilation rate (*F*_*V*)_ is expressed in following way:

(23) $$\begin{array}{r} F_{V}=\frac{u_{i}}{V_{i}\times t}=\frac{\rho \times K_{A}}{t},i\in \left\{1\ldots n\right\} \end{array}$$

for each of *n* analyzed microbial cells or cell fragments.

The error Δ*F*_*V*_ is calculated taking into account the uncertainties of input values (Δρ, Δ*K*_*A*_, Δ*t*_)_ in the following way (Fitzsimons et al., 2000).

$$\begin{array}{l} \Delta F_{V}=\sqrt{{\left(\frac{\partial F_{V}}{\partial \rho }\times \Delta \rho \right)}^{2}+{\left(\frac{\partial F_{V}}{\partial K_{A}}\times \Delta K_{A}\right)}^{2}+{\left(\frac{\partial F_{V}}{\partial t}\times \Delta t\right)}^{2}}\\ \frac{\partial F_{V}}{\partial \rho }=\frac{K_{A}}{t};\frac{\partial F_{V}}{\partial K_{A}}=\frac{\rho }{t};\frac{\partial F_{V}}{\partial t}=-\frac{\rho \times K_{A}}{t^{2}}; \end{array}$$

### Concept application on *P. putida* cells

**Figure 2:**
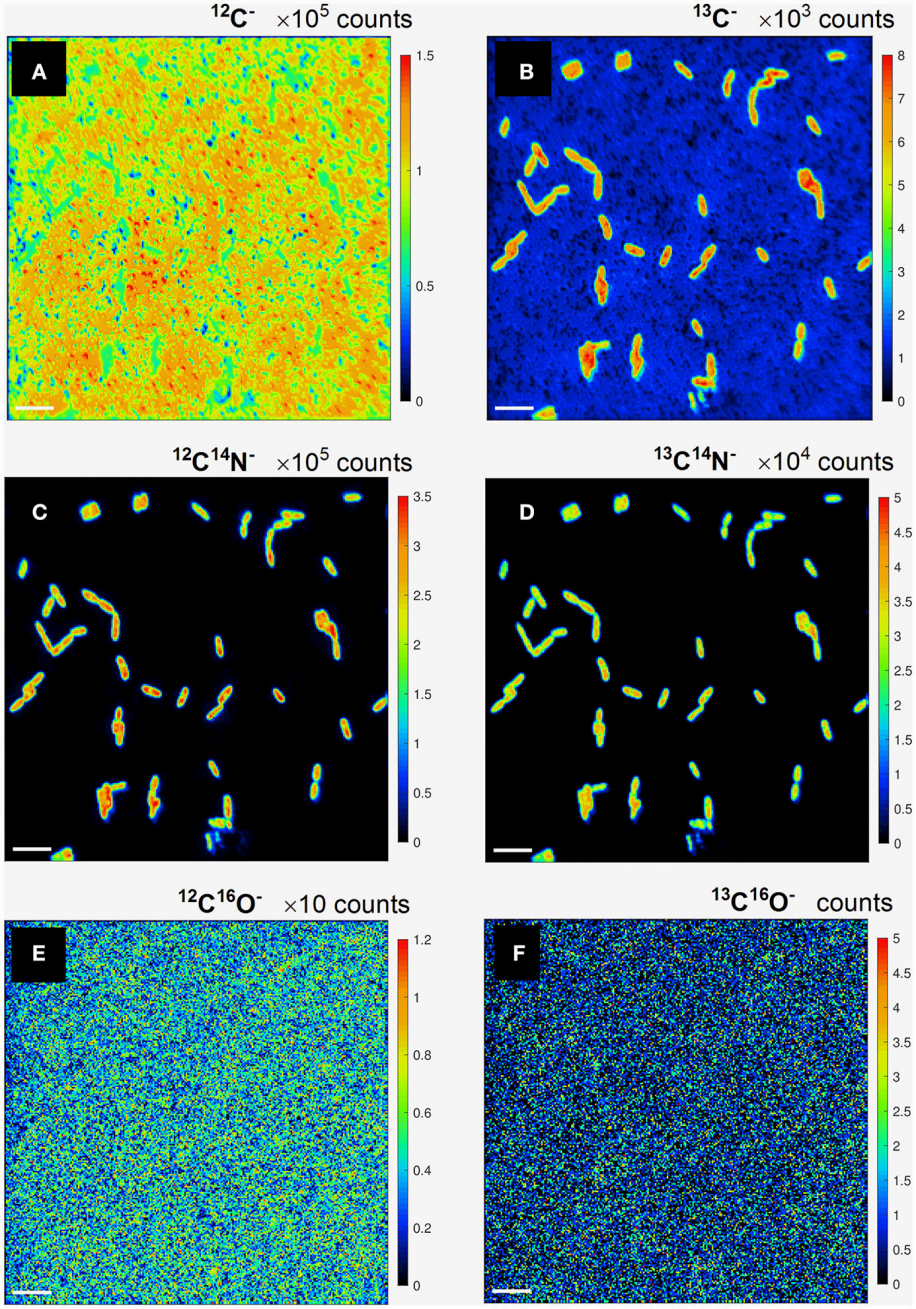
Lateral distribution maps for the relative yield of monoatomic ^12^C^−^ **(a)**, ^13^C^−^ **(b)**, and molecular ^12^C^14^N^−^ **(c)**, ^13^C^14^N^−^ **(d)**, ^12^C^16^O^−^ **(e)**, and ^13^C^16^O^−^ **(f)** secondary ions containing light ^12^C **(a,c,e)** and heavy ^13^C **(b,d,f)** carbon isotopes. Scale bar length is 4 μm.

Carbon assimilation rates were calculated for *P. putida* cells employing the developed quantification procedure using the results of the nanoSIMS experiment. The distribution maps for the relative yield in (i) ^12^C^−^, ^13^C^−^; (ii) ^12^C^14^N^−^, ^13^C^14^N^−^; and (iii) ^12^C^16^O^−^, ^13^C^16^O^−^ secondary ion pairs (Figure 2) were acquired by nanoSIMS. We aimed to derive the carbon isotope ratio (^13^C/^12^C) from both, the counts of monoatomic (C^−^) and molecular (CN^−^ and CO^−^) ions containing different (^12^C and ^13^C) carbon isotopes in order to determine which of these would be more suitable to consider for further calculation of carbon assimilation rates for microbial cells from environmental samples.

The lateral distribution of counts in monoatomic C^−^ ions (Figures 2a,b) reveals a relatively even distribution of ^12^C^−^ counts (Figure 2a) over the analyzed FoVs involving microbial cells and polycarbonate filter substrate, whereas a clear pattern of ^13^C-labeled microbial cell distribution is delivered in ^13^C^−^ counts (Figure 2b). The cell distribution pattern is nicely reproduced with the lateral maps acquired in counts of molecular CN^−^ secondary ions with light and heavy carbon isotopes (Figures 2c,d). Lateral distribution maps of CO^−^ ion counts (Figures 2e,f) as well as their ratio did not revealed any kind of cell distribution pattern and were therefore not considered for further quantitative data evaluation. The detected CO^−^ ion counts were concluded to originate mostly from volatile organic contaminant and residual gas molecules adhered on the sample surface. This adhesion occurs even in ultra-high vacuum (UHV) where residual gas molecules and dust particles are still present. Clarification of unspecific CO^−^ ion origin requires further studies implying detailed analysis of sample molecular composition upon different sample preparation and storage conditions.

**Figure 3:**
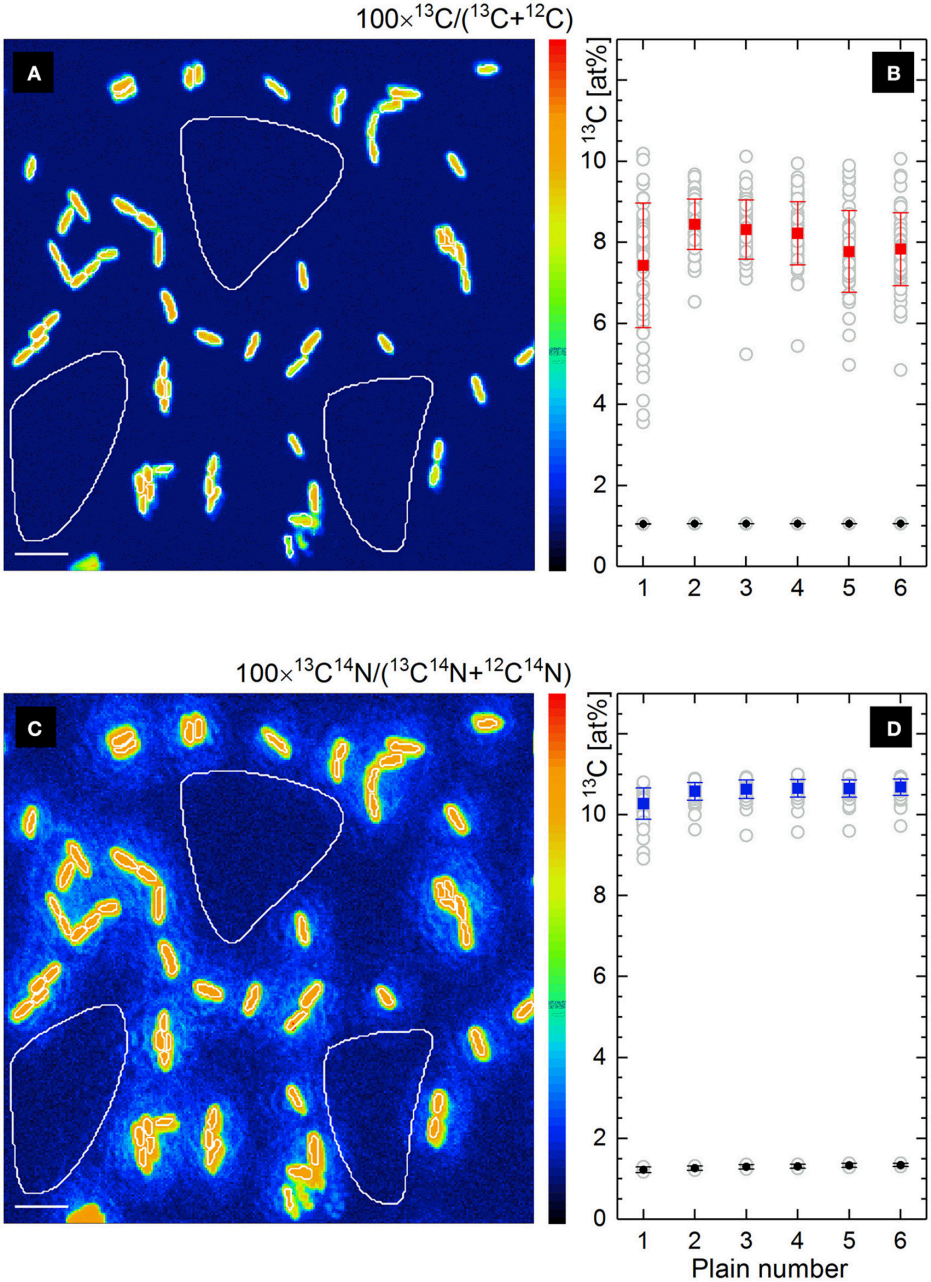
Lateral distribution of ^13^C fraction in at% derived from the isotope ratio of single atomic C^−^ ions **(a)** and molecular CN^−^ ions **(c)** measured by nanoSIMS. Frames ***b*** and ***d*** show the depth profiles of respective ^13^C fractions (gray circles) for all defined RoIs (**R**anges **o**f **I**nterest, white line confined) involving microbial cells and filter areas. The mean value of ^13^C fraction with its standard deviation is shown for cells (solid rectangles) and filter areas (solid circles) in each scanned plain **(b,d)**. Scale bar length is 4 μm.

The maps of ^13^C^−^ and ^13^C^14^N^−^ ion counts (Figures 2b,d) showed a similar distribution and were used to define single cells and reference filter areas (ROIs) by confining of respective cell- or filter-related pixels in freehand drawn loops [(Polerecky et al., 2012); Figures 3a,c, white lines]. More precise and reproducible RoI definition can be done employing the function of “interactive thresholding” (Polerecky et al., 2012) on isotope/ion ratio maps. For a direct comparison of the analysis results the ROIs defined from similarly distributed ^13^C^−^ and ^13^C^14^N^−^ ion counts were used for calculation of ^13^C fraction in monoatomic C^−^ and molecular CN^−^ ions.

Lateral distribution maps of ^13^C fraction derived from the count ratio of monoatomic C^−^ and molecular CN^−^ ions are shown in Figures 3a,c together with the depth profiles of ^13^C fraction over 6 acquired plains (Figures 3b,d) for all defined ROIs.

Contrary to the similar distribution of relative intensity in ^13^C^−^ and ^13^C^14^N^−^ ion count maps (Figures 2b,d), the maps of ^13^C isotope fraction derived from counts of monoatomic C^−^ and molecular CN^−^ ions (Figures 3a,c) were found to be different. The cell size appears to be smaller in ^13^C fraction derived from monoatomic C^−^ ratio (Figure 3a) due to the contribution of ^12^C from filter at the edge of microbial cell. Such a trapping of extrinsic ^12^C from sample substrate (polycarbonate filter) or embedding material into an analyzed RoI area or volume causes a reduction of calculated ^13^C fraction due to the dilution effect. From the monoatomic C^−^ ion ratio the ^13^C fraction of 8.003 ± 0.378 at% has been derived for microbial cells (Figure 3b, solid rectangles) and 1.057 ± 0.003 at% complying with the natural ^13^C abundance for filter areas (Figure 3b, solid circles). Considerably higher mean value of ^13^C fraction with reduced standard deviation (10.585 ± 0.153 at%) have been derived for microbial cells from molecular CN^−^ ion ratios (solid rectangles in Figure 3d). Secondary CN^−^ ions (originating from proteins or nucleic acids) are inherent products of microbial cell ionization and their carbon isotopic composition can be admitted for the whole microbial cell actively growing or grown in environment with a defined isotopic composition. The ^13^C fraction derived from molecular CN^−^ ion ratios for filter areas (1.294 ± 0.045 at%; solid circles in Figure 3d) exceeds the natural ^13^C abundance and the residual CN^−^ ion yield detected from the filter areas may be associated with N-containing cell components (low molecular mass compounds) escaping cells during sample preparation.

The lateral dilution effect observed for the ^13^C fraction in monoatomic C^−^ ratio (Figures 3a,b) may be reduced when RoI definition is based on the C^−^ isotope ratio map by excluding the ^12^C-rich filter material from the RoI area. Deviation in ^13^C fraction derived for microbial cells from isotopic composition of C^−^ (CN^−^) may occur due to the dilution of native cell-specific C^−^ (CN^−^) ions not only with those originating from a sample substrate, but also with C^−^ (CN^−^) from embedding material, from overlapping extracellular organics and other microbial species possessing different ^13^C enrichment. To check for such a spatial dilution in C^−^ (CN^−^) isotopic composition, the depth profile (changes over the scanned planes) of respective (C^−^ or CN^−^) isotope ratio has to be analyzed for each RoI. If a set of planes shows an isotope ratio value which is considerably different from those revealed in other planes of the same RoI, then the planes with close values of isotope ratios have to be accumulated and quantified separately as they are originating from sample compartment with different ^13^C enrichment. Such analysis on complex organic sample is rather complicated due to the ion beam induced intermixing of sample material, sample geometry, its heterogeneity, and surface topography.

#### Reconstruction of original ^13^C fraction in *P. putida* cells

**Figure 4:**
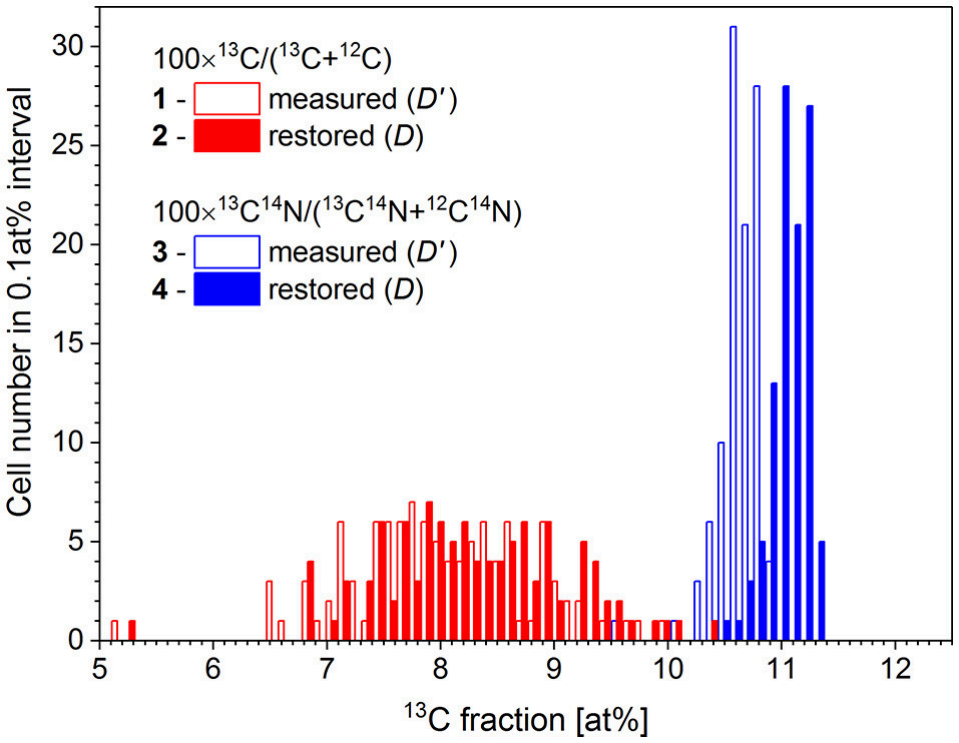
Distribution of microbial cell by the ^13^C fraction derived from isotope ratio of single atomic C^−^ ions (1) and molecular CN^−^ ions (3) as measured with nanoSIMS (*D'*) and the respective distributions (2 and 4) with the ^13^C fraction (*D*) corrected for the dilution of ^13^C label with the chemicals used during cell fixation.

Chemical fixation of *P. putida* cells leads to a 4.4 at% dilution of ^13^C isotope content, relative to the initial cellular carbon content (Musat et al., 2014). With the measured *R'* ratio and a *K* value of 0.044 ± 0.014, the original carbon isotope ratio *R* has been reconstructed for each cell using the expression (8). The ^13^C fraction in chemicals used for cell fixation (*D*_*ch*_) was set at 1.1 at%. The analysis has been done for 100 cells in two FoVs measured with nanoSIMS instrument in the same conditions. The ^13^C fraction *R'* has been calculated for each cell considering the ion counts accumulated in each pixel confined within a corresponding RoI over 6 analyzed planes. The restoration results are shown in Figure 4 and represented in Table 2.

**Table 2:** The values of ^13^C fractions derived for the microbial cells from the measured *R'* and reconstructed *R* isotope ratios of monoatomic C^−^ and molecular CN^−^ ions.

**Considered secondary ions**	**Measured ^13^C fraction *D*' ±Δ*D*' [at.%]**	**Restored ^13^C fraction *D* ±Δ*D* [at.%]**	**Difference *D*- *D'* [at.%]**
Monoatomic C^−^	7.99 ± 0.79	8.29 ± 0.82	0.30
Molecular CN^−^	10.64 ± 0.19	11.06 ± 0.19	0.42

#### Calculation of carbon assimilation rate

**Figure 5:**
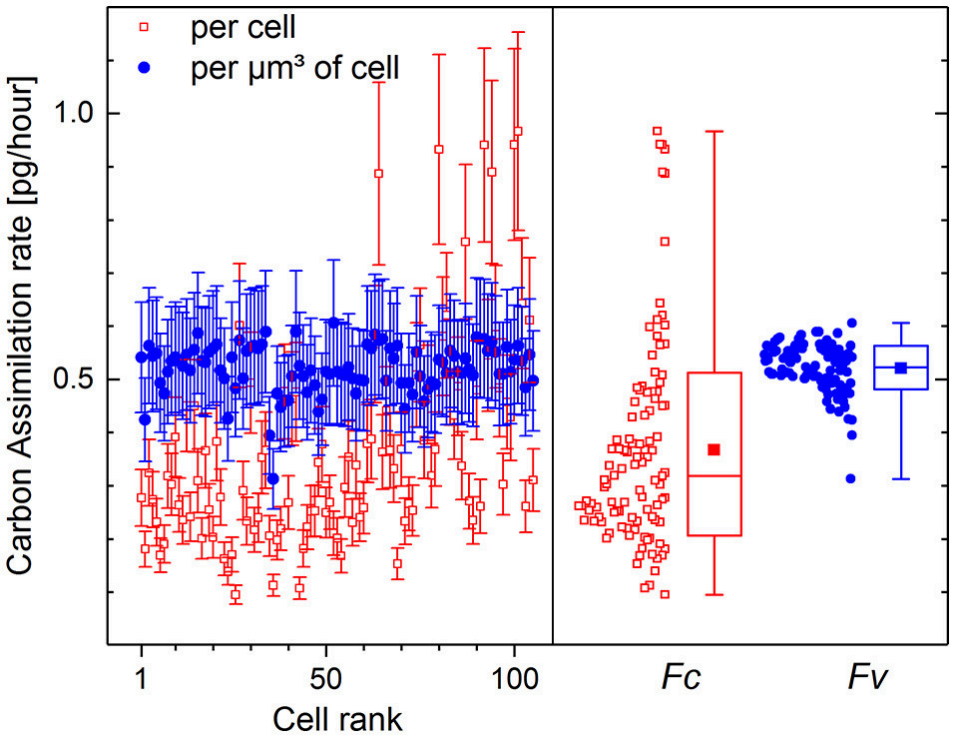
Carbon assimilation rate calculated per volume *F*_*V*_ (solid circles, [pg·μm^−3^·h^−1^]) and per single cell *F*_*c*_ (open rectangles, [pg·cell^−1^·h^−1^]) of *P. putida* incubated in ^13^C-glucose medium (*D*_*gs*_ = 13.5 at%) for 10 h. Initial ^13^C fraction *D*_*i*_ has been set at 1.1 at%. Assimilation rates are shown with mean value and standard deviation for all 105 single cells in the left frame. The distributions of cell-specific (*F*_*c*_) and volume-specific (*F*_*V*_) assimilation rates are shown in the right frame with Min-Max whiskers, box representing the 16–84 percentile range, median value (horizontal line) and mean value (solid rectangle) inside the percentile box.

The values of ^13^C fraction restored from the counts of molecular CN^−^ ions have been chosen for the calculation of carbon assimilation rate. The fraction *K*_*A*_ of carbon incorporated into the cells via biotic assimilation has been calculated for each cell according to (11) with the isotope ratio *R*_*i*_ corresponding to 1.1 at% initial ^13^C fraction (*D*_*i*_) in the microbial cells, *R*_*gs*_ corresponding to 13.5 at% ^13^C fraction (*D*_*gs*_) in growth substrate and the isotope fractionation factor α set to 1. The carbon assimilation rates calculated per cell (*F*_*c*_) according to (22) and per μm^3^ of cell volume (*F*_*V*_) according to (23) are presented in Figure 5.

The cell volume *V*_*i*_ (13) used for the calculation of *F*_*c*_ (22) was derived from the size and geometry of RoIs that are supposed to be drawn around single microbial cells, but are often drawn around their visualized fragments of different sizes. The variation of RoI size affects the estimated cell volume and results in strong dispersion of carbon assimilation rate values delivering 0.37 ± 0.19 pg·cell^−1^·h^−1^ as calculated per microbial cell (Figure 5, open rectangles). The volume-specific carbon assimilation rate *F*_*V*_ (23) calculated per μm^3^ of single cell volume shows considerably lower dispersion (Figure 5, solid circles) delivering 0.52 ± 0.04 pg·μm^−3^·h^−1^.

## Conclusions, applicability, and critical considerations

Here we provide a comprehensive model to derive assimilation rates of single cells from SIP-nanoSIMS experiments. Particular features of our model include careful consideration of both physiological (isotope fractionation during substrate uptake) and experimental (sample preparation applied) effects on the isotopic composition of cells. In addition we provide detailed considerations and recommendations to determine the cellular density of carbon, which could also be applied to obtain the cellular density of other elements as well, for example N, P, or O.

Application of our concept on a model culture showed that when isotope dilution due to various sample preparation methods, or substrate-specific isotope fractionation factors are not considered, assimilation rates can be significantly underestimated. In addition, we observed that even upon normalization of the assimilation rates by cellular volume, *P. putida* cells still showed a relatively high functional heterogeneity. Since the cells were grown with a soluble substrate, constant mixing, and were collected in their mid-exponential growth phase, it is unlikely that heterogeneity was caused by diffusion limitations of oxygen or growth substrate. An attractive hypothesis is that the heterogeneity could be a response of cell physiology to depletion of substrate concentration in a closed system. The heterogeneity and relative difference in cell activity can be represented with the distribution of *K*_*A*_ values showing the relative assimilation of single cells.

The calculation method presented here considers the uncertainties of all input parameters (Δ*R*, Δ*K*, Δ*D*_*ch*_, Δρ, Δ*D*_*gs*_, Δ*D*_*i*_, Δα, Δ*t*) propagating into the error of relative assimilation (*K*_*A*_), volume-specific (*F*_*V*_), and cell-specific (*F*_*C*_) assimilation rates expressed for each single cell. The error consideration is particularly important for the input values used in calculations on single-cell level (i.e., fraction *K* of carbon introduced via chemical treatment, element-specific cellular density, e.g., ρ_*C*_ or ρ_*N*_) but derived on a bulk level for similar phylotypes. The demand of element-specific cellular density (ρ) is an inherent shortcoming of the single-cell assimilation rate quantitation when it is applied to environmental populations, where the ρ values of individual cells are unknown and have to be approximated with an average value derived for a pure culture or for an environmental bulk population. Although the approximation of element-specific cellular density introduces an uncertainty in the calculated rates, no viable alternative is available to date. Consideration of an element-specific cellular density (e.g., ρ_*C*_ or ρ_*N*_) estimated using the Loferer-Krossbacher approach (Equation 15) and Redfield elemental ratio (e.g., Equation 17) may also cause significant errors in the calculated volume- and cell-specific assimilation rates (*F*_*V*_ and *F*_*C*_). If the value of element-specific cellular density (ρ) is unknown and its approximation is considered to cause an unacceptable uncertainty in the calculated assimilation rates, then assimilation can be expressed relatively with *K*_*A*_ values (Equation 11) derived without ρ consideration.

Calculation of cell-specific assimilation rate (*F*_*C*_, Equation 22) involves the RoI-confined volume of each single cell (*V*_*i*_) that may result in uncertainty and artificial dispersion of *F*_*C*_ values when not entire cells but rather cell fragments are confined within the defined RoIs. The calculation of volume-specific assimilation rate (*F*_*V*_, Equation 23) may be a viable solution in this case. The *F*_*V*_ values are independent from the RoI-confined cellular volume and can be considered together with biovolume and cell abundance in specific environmental microbial populations for upscaling the assimilation rate from single-cell to ecosystem level. For example, one can quantify the role of certain microbial populations in specific biogeochemical processes, like nitrogen fixation in oligotrophic waters (Thompson et al., 2012; Martínez-Pérez et al., 2016). This type of quantitative data, previously inferred from bulk measurements and cell counts or sequence abundances, were often underestimating the role of key players and their significance in the environment. Nowadays, the quantitation of assimilation rate at single-cell level in complex microbial communities cannot be performed without SIP-FISH-nanoSIMS technique and the suggested calculation method has therefore an inevitable applicability in many experimental as well as environmental studies.

## Author contributions

NM, HR, and HS conceived the study and the experimental design. NM and FC performed the cell incubation and sampling. FC and SK executed the elemental density analysis. FC and HS performed the nanoSIMS analysis. HS developed the mathematical model development and the first draft of the manuscript. HS, NM, and FM wrote the manuscript with contribution from all authors. All authors contributed to manuscript revision, read and approved the submitted version.

### Conflict of interest statement

The authors declare that the research was conducted in the absence of any commercial or financial relationships that could be construed as a potential conflict of interest.
