# Supplementary material for: Calculation of Single Cell Assimilation Rates From SIP-NanoSIMS-Derived Isotope Ratios: A Comprehensive Approach
Source: Front Microbiol. 2018 Oct 3;9:2342. doi: 10.3389/fmicb.2018.02342 (PMC6178922; doi:10.3389/fmicb.2018.02342)
Supplement: Supplementary file 2 [file Data_Sheet_1.PDF]

*Supplementary Material*

# Calculation of single cell assimilation rates from SIP-nanoSIMS-derived isotope ratios: a comprehensive approach

**Hryhoriy Stryhanyuk*, Federica Calabrese, Steffen Kümmel, Florin Musat, Hans H. Richnow and Niculina Musat***

*** Correspondence:**
Hryhoriy Stryhanyuk; Niculina Musat
gregory.stryhanyuk@ufz.de; niculina.musat@ufz.de

## 1 Supplementary Data

### 1.1 SEM imaging of *P. putida* cells for their biovolume determination

The biovolume values are usually determined by measuring the cell dimensions and calculating the volume of the geometrical figure confining the cell. In the present study, the rod and coccoid cell shape were considered. The cell dimensions were measured on images acquired with Scanning Electron Microscope (SEM). An example of SEM image from a section of a filter loaded with *P. putida* cell is shown in Fig. S1. Several SEM images were used for measurements of cell dimensions (length and width). The length and width of every single cell was measured using ImageJ software. *P. putida* cell was considered as a cylinder with two hemispherical extremities upon biovolume calculation.

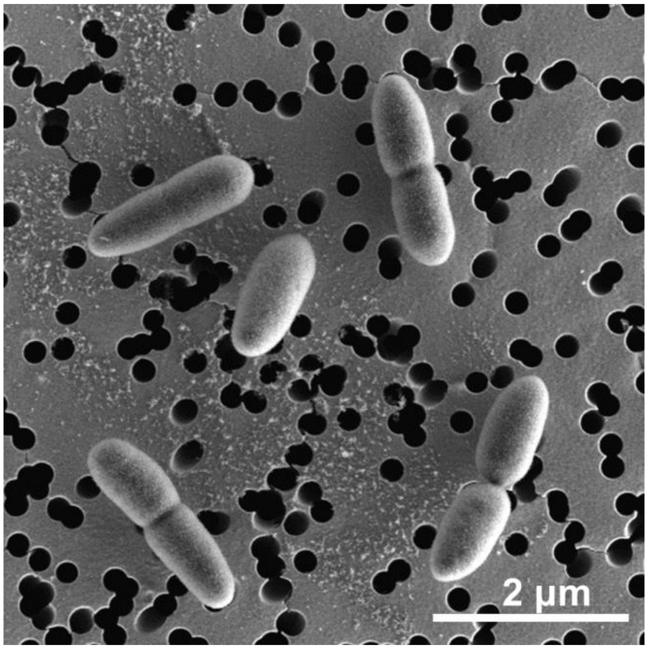


**Figure S1**. SEM micrograph of *Pseudomonas putida* cells.

The obtained values of length and width were 1.038 ± 0.138 and 0.568± 0.037 µm, respectively, and were used to derive the biovolume value of 0.22±0.06 µm$^3$.

## 1.2 Consideration of Isotope fractionation

Many biochemical processes are associated with isotope fractionation leading to separation of the isotope composition between substrate and product. For example, the assimilation of biological elements such as carbon, nitrogen or hydrogen is often governed by isotope fractionation which has its origin in the kinetic isotope effect of a biochemical reaction discriminating between isotopologues. Thus the isotope fractionation upon the assimilation of substrates used for biosynthesis should be considered for the calculation of substrate transformation into the biomass with respect to accumulation efficiency and substrate turn-over. The specific isotope fractionation associated with biosynthesis of a particular process resembles a number of isotope effects from subsequent biochemical reactions and is often difficult to assess precisely. In addition, the isotope fractionation may be dependent on biochemical pathway, physiology, growth conditions and others. Thus a broad estimation of the isotope fractionation processes could be made by using (i) empirical factors reported for particular biological reactions, (ii) fractionation factors reported for biological processes or (iii) semiclassical Streitwieser limits (Huskey W.P. "Origin and Interpretation of heavy-atom Isotope effects." In: Cook, P. F. "Enzyme mechanism from isotope effects". CRC Press: 1991. pp 37-72. ISBN: 0-8493-5312-2) that are the kinetic isotope effects expected for a particular bond cleavage reaction. The latter approach may be used to characterize the upper limit of isotope effects during assimilation which may not be expected in real biological experiments but may be useful to estimate the maximal effect of isotope fractionation.

The rational for refining the assimilation of substrate in labelling experiments is to correct for kinetic isotope effect from a series of biochemical reactions using kinetic isotope fractionation factors to account for isotope effects in mass balances and finally for quantification of assimilation/activity in biological system. The kinetic isotope fractionation factor ($\alpha$) is used to account for the isotope fractionation.

For illustration how isotope fractionation affecting the labelling of biomass we compare 3 scenarios. In the first scenario the isotope fractionation factor ($\alpha$) is set to 1 meaning no isotope fractionation. In the second scenario we select the Streitwieser limit for a C-C bond cleavage ($\alpha_C$ =1.048) which may represent almost the upper limit for a possible carbon isotope fractionation. In the third scenario we consider the value $\alpha$=1.026 reported for $CO_2$ as a result of organic material respiration in soil (Martin Alexander (1994) "Biodegradation and bioremediation". San Diego: Academic Press. ISBN: 0-12-049860-X).

The calculations of assimilation rate reported in present studies were performed with the isotope fractionation factor $\alpha$ set to 1 and compared to $\alpha$ >1. To elucidate the extent of isotope fractionation during the isotope labelling of microbial cells upon metabolism, the expression describing the relation between $^{13}$C fractions $D_1$ achieved with $\alpha$ =1 and $D_2$ achieved with $\alpha$ >1 has been derived with corresponding $R$ values of carbon isotope ratio in the following way.

$$\boldsymbol{D_1 = \frac{R_1}{R_1 + 1} \Rightarrow R_1 = D_1 R_1 + D_1 \Rightarrow R_1 - D_1 R_1 = D_1 \Rightarrow R_1 \times (1 - D_1) = D_1}$$

$$R_1 = \frac{D_1}{(1 - D_1)}$$

$\alpha$ is the isotope fractionation factor describing the fractionation between substrate and biomass.

$$\begin{cases} D_2 = \frac{R_2}{R_2 + 1} \\ \alpha = R_1/R_2 \;\Rightarrow\; R_2 = R_1/\alpha \end{cases} \Rightarrow \; D_2 = \frac{R_1/\alpha}{R_1/\alpha + 1} = \frac{R_1}{R_1 + \alpha}$$

$$\begin{cases} D_2 = \frac{R_1}{R_1 + \alpha} \\ R_1 = \frac{D_1}{(1 - D_1)} \end{cases} \Rightarrow \; D_2 = \frac{\frac{D_1}{(1 - D_1)}}{\frac{D_1}{(1 - D_1)} + \alpha} = \frac{D_1}{D_1 + \alpha \times (1 - D_1)} \Rightarrow$$

$$D_2 = \frac{1}{1 + \alpha \times \left(\frac{1}{D_1} - 1\right)}$$

The fraction $D_2$ considers the isotope fractionation in comparison to $D_1$ calculated without taking isotope fractionation into account (with $\alpha$=1.000). The cellular $^{13}C$ fractions ($D_1$) of individual cells is derived in nanoSIMS experiment from $^{13}C^{14}N/^{12}C^{14}N$ ratio of molecular $CN^-$ ions and corrected for the dilution of $^{13}C$ label by the chemicals used for cell fixation (Fig. S2 (blue bars).

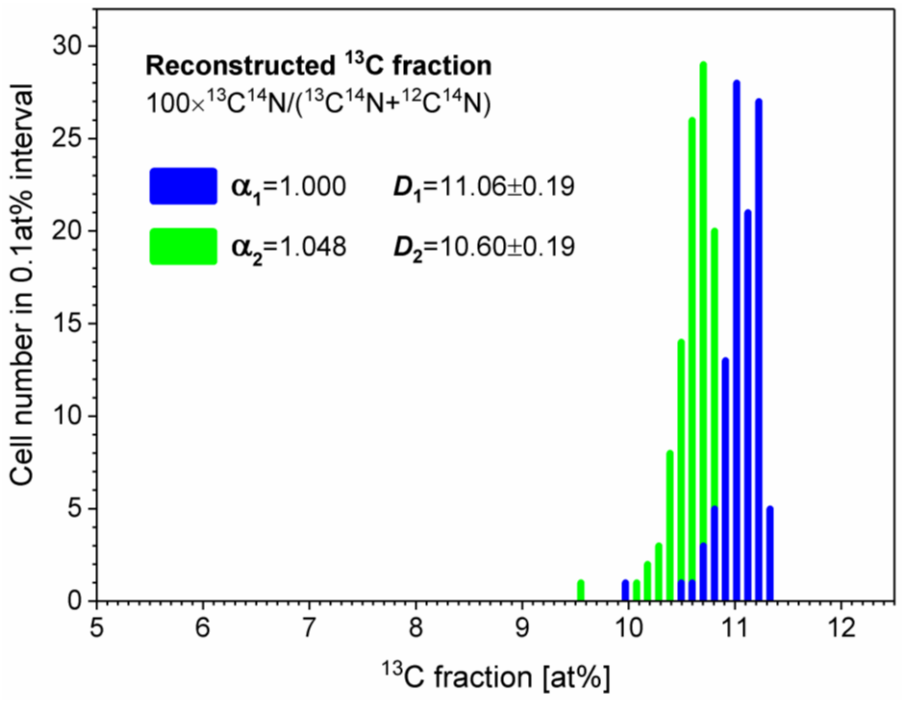


**Figure S2**. Distributions of $^{13}C$ labelled cells. The $^{13}C$ isotope fraction $D_1$ derived after nanoSIMS experiment assuming a kinetic isotope fractionation factor $\alpha$=1.000 (blue bars) and reconstructed $D_2$ with $\alpha$=1.048 (green bars).

The distribution of nanoSIMS-derived cellular $^{13}$C fraction $D_1$ (Fig. S2 (blue bars) is compared with the distribution of $^{13}$C fraction $D_2$ reconstructed assuming isotope fractionation processes ($\alpha$=1.048) due to the discrimination of carbon isotopes upon assimilation which causes a decrease in $^{13}$C incorporation (Fig. S2 (green bars).

Thus, isotope fractionation affects the final labeling in $^{13}$C as illustrated (Fig S2) because light carbon isotope is preferred during assimilation when $\alpha$>1. In the example we calculate the lowering of the labeling ($D_2$) using the isotope fractionation factor $\alpha$=1.048 corresponding to semiclassical Streitwieser limits (the value has been taken from Table 1 in (Huskey W.P. "Origin and Interpretation of heavy-atom Isotope effects." In: Cook, P. F. "Enzyme mechanism from isotope effects". CRC Press: 1991. pp 37-72. ISBN: 0-8493-5312-2).

Taking into account the effect of isotope fractionation factor $\alpha$=1.048 (Fig. S2) the calculations of volume-specific assimilation rate $F_V$ and fraction of assimilated carbon $K_A$ were performed for different values of isotope fractionation factor ($\alpha$) within 1.000-1.050 range. The resulted dependences of $F_V(\alpha)$ and $K_A(\alpha)$ are plotted in Fig. S3.

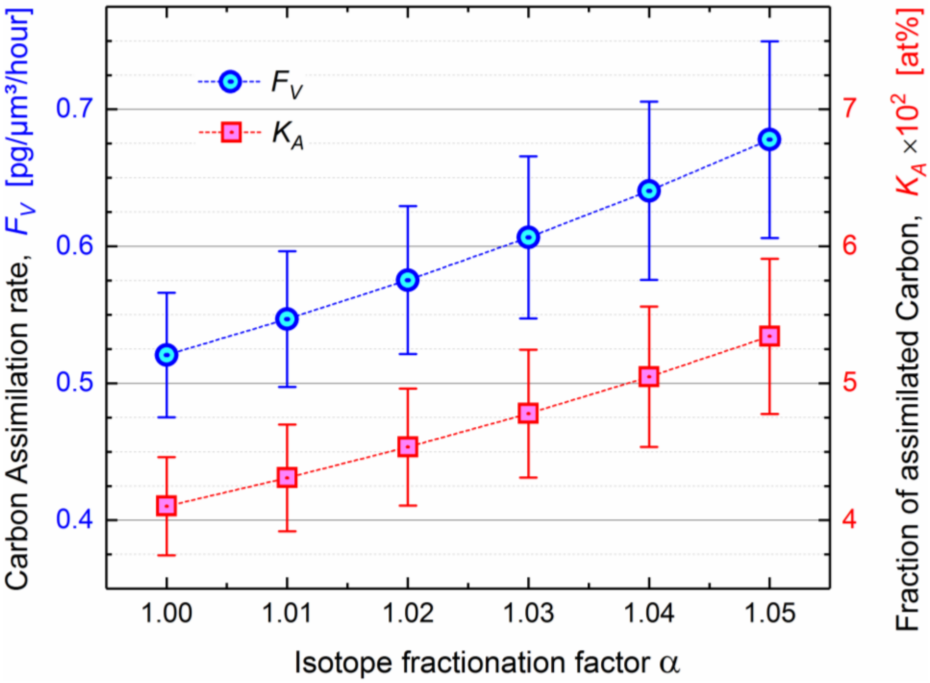


**Figure S3**. Volume-specific assimilation rate $F_V$ and fraction of assimilated carbon (relative assimilation) $K_A$ calculated for different isotope fractionation factors ($\alpha$). The mean values and standard deviation (± 1σ error bars) of volume-specific assimilation rate ($F_V$) and relative assimilation ($K_A$ fraction of assimilated carbon relative to its initial cellular content) are shown.

To illustrate the isotope fractionation effect during assimilation we have analyzed also the difference in the relative assimilation calculated with and without consideration of isotope fractionation for different $^{13}$C fraction in growth substrate ($D_{gs}$). The difference has been calculated as a relative error ($\Delta K_A$) using the following expression.

$$\Delta K_A = \frac{(K_A(\alpha > 1) - K_A(\alpha = 1))}{K_A(\alpha = 1)} \times 100\ [\%]$$

The calculated $\Delta K_A$ ($D_f$) dependences are plotted in Fig. S4 for different fraction of $^{13}$C in growth substrate assuming the isotope fractionation factors $\alpha$=1.048 (semiclassical Streitwieser limit, dotted lines) or $\alpha$=1.026 (solid lines). The relative error in the calculation can be substantial and may reach the order of a calculated $K_A$ value for the transformation when the labeling of the cell ($D_f$) approach the $^{13}$C fraction in the growth substrate ($D_{gs}$).

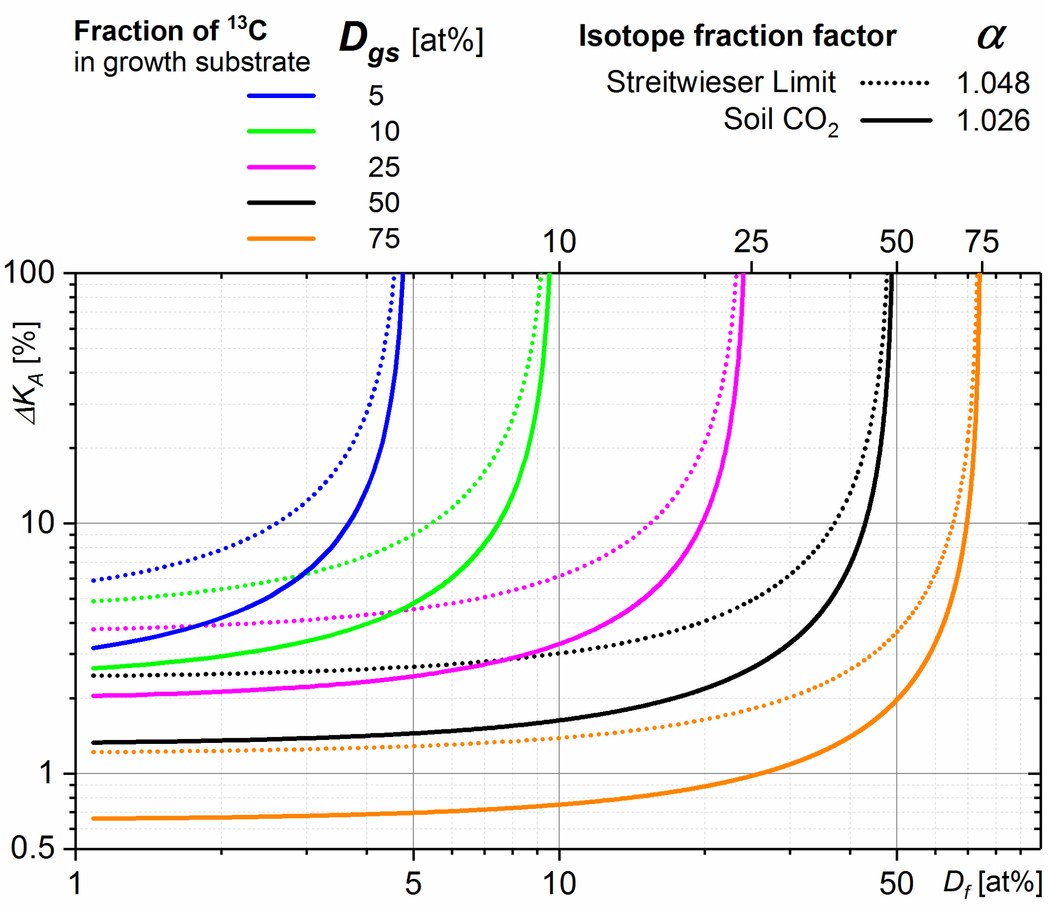


**Figure S4**. Dependence of the relative error $\Delta K_A$ for the assimilated carbon fraction calculated for different fractions of $^{13}$C in growth substrate ($D_{gs}$) with $\alpha$=1 neglecting isotope fractionation. Initial fraction of $^{13}$C in cell inoculum is 1 at%. The relative error is presented for the cases of ignoring the isotope fractionation factors $\alpha$=1.026 (solid lines) and $\alpha$=1.048 (dotted lines) upon the calculation of assimilation with the fractionation factor $\alpha$=1.000.

The value $\alpha$=1.026 has been calculated with $\delta^{13}$C=-0.025 reported for $CO_2$ in soil as a result of organic material respiration (-25‰ derived from Fig. 5-1 in (Martin Alexander (1994) “Biodegradation and bioremediation”. San Diego: Academic Press. ISBN: 0-12-049860-X) using the following expression linking $\alpha$ and $\delta^{13}$C ($\delta$).

$$\alpha = \frac{1}{(\delta + 1)}$$

The $\Delta K_A$ value increases when $D_f$ approaches $D_{gs}$ and is especially important for consideration at low $D_{gs}$ values.

## 2 Comparison of $K_A$ expression with the expression of net assimilation $Fx_{net}$ reported by Popa et al. 2007

Expression for the net assimilation $Fx_{net}$ from Popa et al. 2007 (copied from article PDF):

$$\mathrm{Fx_{net}} = \{R_f[1 - R_i/(R_i + 1)] - R_i/(R_i + 1)\}/\{R_s/(R_s + 1) - R_f[R_s/(R_s + 1)]\} \times 100\% . \quad (6)$$

Taking the following denotations into account

$$R_s \equiv R_{gs}$$

$R_{gs}$ – isotope ratio in labelled growth substrate;
$R_i$ – initial cellular isotope ratio before incubation;
$R_f$ – cellular isotope ratio after incubation

$$\boldsymbol{Fx_{net}} = \frac{\boldsymbol{R_f} \times \left[\boldsymbol{1} - \frac{\boldsymbol{R_i}}{\boldsymbol{R_i} + 1}\right] - \frac{\boldsymbol{R_i}}{\boldsymbol{R_i} + 1}}{\frac{\boldsymbol{R_{gs}}}{\boldsymbol{R_{gs}} + 1} - \boldsymbol{R_f} \times \frac{\boldsymbol{R_{gs}}}{\boldsymbol{R_{gs}} + 1}}$$

It can be also transformed in the following way

$$\boldsymbol{Fx_{net}} = \frac{\boldsymbol{R_f} - [\boldsymbol{R_f} + \boldsymbol{1}] \times \frac{\boldsymbol{R_i}}{\boldsymbol{R_i} + 1}}{[\boldsymbol{1} - \boldsymbol{R_f}] \times \frac{\boldsymbol{R_{gs}}}{\boldsymbol{R_{gs}} + 1}}$$

In our work we have used the same model of two component mixing as reported by Popa et al. 2007 and we obtained a bit different expression.

$$\boldsymbol{K_A} = \frac{\boldsymbol{E_a}}{\boldsymbol{E_i}} = \frac{\boldsymbol{R_f} - \boldsymbol{R_i}}{\boldsymbol{R_i} + 1} \times \frac{\boldsymbol{R_{gs}} + 1}{\boldsymbol{R_{gs}} - \boldsymbol{R_f}}$$

The last two expressions ($F\mathrm{x_{net}}$ and $K_\mathrm{A}$) are suggested to describe the amount of assimilated carbon relative to its initial cellular content in previous report (Popa et al. 2007) and our work. Both are using similar parameters and definitions.

$$F\mathrm{x_{net}} \equiv K_A$$

To estimate the difference between $F\mathrm{x_{net}}$ and $K_\mathrm{A}$ we plotted their dependences on $D_f$ (see Fig. S5) using the final expression for $K_\mathrm{A}$ derived in our work (Eq. 11)

$$K_A = \frac{R_f - R_i}{(1+R_i) \times \left[\frac{R_{gs}}{R_{gs}+\alpha} \times (1+R_f) - R_f\right]}$$

with $\alpha$=1 and

$$R_f = \frac{D_f}{1-D_f}$$

The plot of $Fx_{net}(D_f)$ dependences calculated according to Popa et al. 2007 shows an agreement with our calculations only when $D_{gs}$=0.5. Actually, the $Fx_{net}(D_f)$ dependence reveals an asymptote at $D_f$=50 at% for all values of $^{13}C$ fraction in growth substrate ($D_{gs}$) that is unreasonable. On one hand, cells cannot reach a $^{13}C$ fraction exceeding ($D_{gs}$) without extreme inverse isotope fractionation. On the other hand, their enrichment should not be limited to 50 at% of $^{13}C$ if higher $^{13}C$ fraction is available in growth substrate. The difference between $Fx_{net}$ and $K_A$ increases with $D_f$ and is especially considerable for lower $D_{gs}$ values.

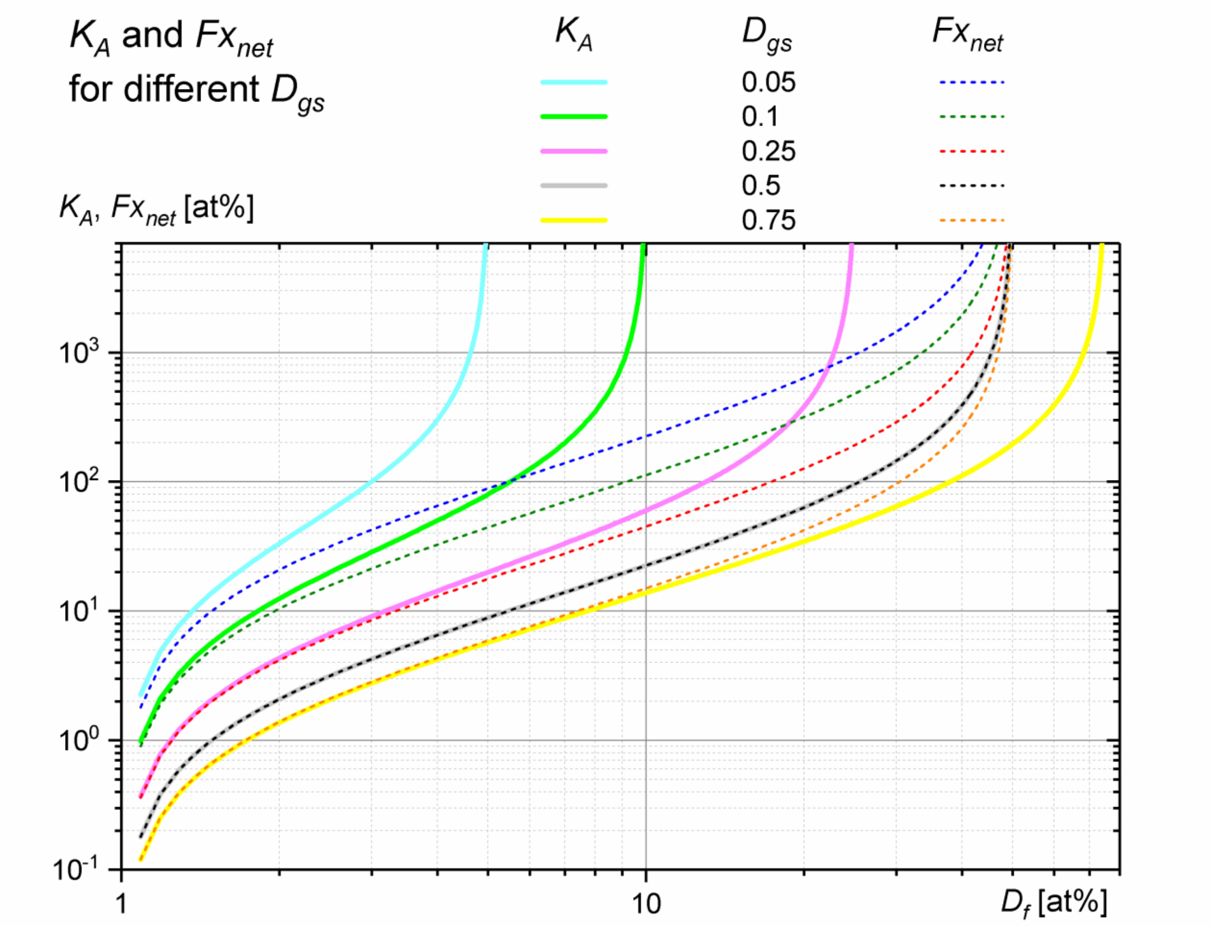


**Figure S5**. Dependence of the assimilated carbon fraction expressed as $K_A$ (***solid***, this work) with $\alpha$=1 and as $Fx_{net}$ (***dashed***, Popa et al. 2007) on the final cellular $^{13}C$ fraction $D_f$ simulated for the cells incubated with the inoculum with $D_i$=1 at% (initial $^{13}C$ fraction in cells) in the growth substrates with different $D_{gs}$ values of $^{13}C$ fraction.

## 3 Supplementary Table

Generalized scheme of an experiment optimized for assimilation rate studies

| | | | |
|---|---|---|---|
| 1. | Incubation in isotope-labelled growth substrate<br>- cell sampling (for EA-MS with cell counting and nanoSIMS) at several time points within their exponential growth phase<br>- sampling of growth substrate and measurements of $D_{gs}$ for each sampling point | | |
| 2. | Cell fixation and CPD | | |
| 3. | Estimation of cell volume from SEM or AFM experiment.<br>*The derived mean value of cell volume is used further for calculation of element-specific cellular density.* | | |
| 4. | Derivation of element-specific cellular density from an Elemental Analysis (EA) and cell counting experiments (Eq. 14) using the cell volume derived at Step 3 | or | Calculation of element-specific cellular density using the Loferer-Krossbacher approach (Eq. 15), Redfield elemental ratio, EA data and the cell volume derived at Step 3 |
| 5. | nanoSIMS measurements and data evaluation providing isotope ratio $R'$ for each sampling point | | |
| 6. | Restoration of isotope ratio $R$ using expression (8) considering the measured $R'$ isotope ratio, the fraction $K$ of carbon introduced into a cell during chemical treatment and fraction $D_{ch}$ of heavy isotope in the chemicals. | | |
| 7. | Calculation of $K_A$ fraction of carbon or nitrogen incorporated into the cells via assimilation using Eq. 11 | | |
| 8. | Calculation of cell- or volume-specific assimilation rate using the Eq. 22 or Eq. 23 | | |

*Steps 6-8 are implemented in the supplementary Excel template table
